# Small Molecule Agents Targeting PD-1 Checkpoint Pathway for Cancer Immunotherapy: Mechanisms of Action and Other Considerations for Their Advanced Development

**DOI:** 10.3389/fimmu.2022.752065

**Published:** 2022-05-02

**Authors:** Pottayil G. Sasikumar, Murali Ramachandra

**Affiliations:** Aurigene Discovery Technologies Limited, Bangalore, India

**Keywords:** PD-L1 inhibitors, cancer immunotherapy, mechanism of action (MOA), small molecule immunomodulators, small molecule PD-1/PD-L1 inhibitors

## Abstract

Pioneering success of antibodies targeting immune checkpoints such as programmed cell death protein 1 (PD-1) and cytotoxic T-lymphocyte-associated protein 4 (CTLA-4) has changed the outlook of cancer therapy. Although these antibodies show impressive durable clinical activity, low response rates and immune-related adverse events are becoming increasingly evident in antibody-based approaches. For further strides in cancer immunotherapy, novel treatment strategies including combination therapies and alternate therapeutic modalities are highly warranted. Towards this discovery and development of small molecule, checkpoint inhibitors are actively being pursued, and the efforts have culminated in the ongoing clinical testing of orally bioavailable checkpoint inhibitors. This review focuses on the small molecule agents targeting PD-1 checkpoint pathway for cancer immunotherapy and highlights various chemotypes/scaffolds and their characterization including binding and functionality along with reported mechanism of action. The learnings from the ongoing small molecule clinical trials and crucial points to be considered for their clinical development are also discussed.

## Background

Checkpoint inhibitors have transformed cancer therapy by harnessing the power of the immune system to fight cancer, and this breakthrough has now been considered as one of the most exciting discoveries of the twenty-first century (1). Among the various cancer immunotherapies such as checkpoint inhibitors, adoptive T-cell transfer, oncolytic viruses and cancer vaccines, immune checkpoint inhibitors have shown remarkable response in clinical trials and are currently regarded as the most successful class of cancer immunotherapy. Since the Food and Drug Administration (FDA) approval of anti-CTLA-4 antibody ipilimumab in 2011, several antibodies targeting PD-1/programmed death-ligand 1 (PD-L1) immune checkpoint pathway have been approved for cancer therapy in various indications with many more in the pipeline (2). In this review, we have highlighted the progress in the discovery and development of small molecule agents interfering in the PD-1 pathway, with majority of the reported compounds targeting PD-L1, along with their mechanisms of action and specific considerations that are relevant for their advanced development.

## Limitations of Immune Checkpoint Blockade Therapy Associated With Antibodies

While these antibody-based therapies show notable clinical activity, they suffer from serious treatment-related toxicities known as immune-related adverse events (irAEs) mostly due to the dysregulation in the immune system balance (3). The wide range of irAEs are reported to involve almost any tissue or organ with most severe complications manifesting as skin rashes, pneumonitis, hypothyroidism, pancreatitis, encephalopathy, hepatitis, myocarditis, and immune cytopenias (4). Antibodies targeting PD-1 pathways are reported to have lower incidence of adverse events than agents targeting CTLA-4 pathway (5), whereas combination therapy with antibodies targeting both CTLA-4 and PD-1 is reported to have higher rate of irAEs with a greater number of grade 3 and 4 treatment‐related adverse events and treatment discontinuations (6, 7). Even though irAEs can be resolved by appropriate management of immunosuppression with corticosteroids or other immunosuppressant agents such as infliximab, it may expose patients to a higher risk of developing infections (8). Sustained target inhibition due to long half-life (>15–20 days) and ~70% target occupancy for months are likely contributing to severe irAEs (9–11). Apart from toxicity, one of the major deficiencies of approved PD-1/PD-L1 targeted antibodies is their response only in a subset of patient population, which could be partly due to the compensatory mechanisms such as upregulation of alternative immune checkpoints such as T-cell immunoglobulin and mucin-domain containing-3 (TIM-3) and V-domain Ig suppressor of T-cell activation (VISTA) (12, 13). Physiological barriers of antibodies (14) limit their tumor exposure, and the large size of these agents warrants their intravenous dosing in a hospital setting. Last but not the least is the low affordability, since the treatment cost for a single agent therapy can reach more than US$100,000 per patient annually. Furthermore, the requirement for the rational combination with other therapeutic agents to achieve greater response is expected to make checkpoint antibody therapy prohibitively expensive (15).

## Opportunities for Small Molecule Agents to Address the Limitations

Even though deficiencies of antibody-based PD-1/PD-L1 targeted agents underscore the need for alternate approaches, the development of small molecule inhibitors has been significantly behind despite the great potential. The advantages of small molecule agents over antibodies to target PD-1 and other immune checkpoint pathways are summarized in **Table 1**. However, small molecule agents also have a few limitations including their shorter half-life, broad drug distribution resulting in on- and off-target toxicity, potential for reduced specificity and selectivity, and species-specific activity in some instances, making it highly challenging to find appropriate preclinical pharmacology models. These shortcomings might have contributed to the initial lack of enthusiasm in the scientific community compared to monoclonal antibody-based inhibitors as reflected in dramatically less preclinical and clinical efforts focused on the small molecule-based approach. Lessons learned from the highly successful development of small molecule therapeutics against specific targets including protein tyrosine kinases, growth factor receptors, and cell cycle regulatory proteins can be adapted to fully exploit the distinct advantages of small molecule approaches (**Table 1**).

**Table 1:** Advantages of small molecule agents over antibodies to target PD-1 and other immune checkpoint pathways.

Parameter	Antibody	Small molecule
Route of administration	Requirement to dose by intravenous or other parenteral route making it inconvenient to patients (16) Administration in a clinical setting adds to the cost	Potential for oral bioavailability offering the convenience to patients No need to visit the clinic for dose administration (17)
Tumor distribution	Limited tumor distribution because of the larger size (18)	Better tumor distribution expected and greater opportunities to fine tune the physico-chemical properties for improvement
Clinical response	Due to high degree of selectivity, response not expected when intrinsic and adaptive resistance are due to another immune checkpoint pathway(s) (12, 13)	Simultaneous targeting of more than one immune checkpoint proteins with the same agent because of the significant structural homology to drive greater response possible (19, 20)
Management of immune-related toxicity	Due to long pharmacokinetic half-life, typically in weeks, aggressive treatment with immunosuppressants needed while increasing the infection risk (8)	Because of the shorter pharmacokinetic half-life, typically in hours, treatment cessation can be employed for better management of any emergent adverse events (21)
Manufacturing and logistics cost	Due to recombinant mode of production, high cost associated with production, product heterogeneity, and greater regulatory hurdles (18) Cold chain transport and storage required because of their thermo labile nature	Synthetic mode of production leading to lower cost of goods, product homogeneity, and lower regulatory hurdles (22) Cold chain transport and storage not needed due to their thermo stability
Regulatory hurdles	High due to recombinant production (23, 24)	Low and straight forward due to synthetic production

## Considerations for Targeting PD-1 Signaling Pathway Using Small Molecules

The PD-1–PD-L1 receptor–ligand interaction is a classic example of protein–protein interaction (PPI); hence, designing inhibitors for these interactions are highly challenging. Antibodies and fusion proteins are the preferred approach to modulate such PPI dysregulation primarily due to (a) the large interfacial area of the interaction (1,500–3,000 Å), (b) the presence of flat interface without deep and well-defined pockets that are suitable to bind a ligand with high-affinity, (c) the lack of endogenous low molecular ligands that can be considered as reference standards for small molecule chemistry starting points (25, 26)

General perception is that the druggability of an interaction pocket increases logarithmically with total surface area and non-polar contact area, while it decreases logarithmically with polar contact area (27). Efforts to increase the affinity of small molecules to the binding partner often results in increased lipophilicity, which is believed to negatively impact the druggability due to reduced solubility, bioavailability, and increased off-target toxicity. Natural complexes, either protein–protein interactions or protein–peptide ligand interactions, typically engage in more polar contacts than synthetic greasy molecules, with a lot of unmatched hetero atoms bound to proteins. Druggable binding sites are often oversimplified as closed, hydrophobic cavities, but data set analysis reveals that polar groups in druggable binding sites have properties that enable them to play a decisive role in ligand recognition (28). The influence of hotspots, which are small areas of the protein–protein interface contributing most of the binding energy within one hot region, is cooperative to stabilize protein interfaces, and they are networked to provide stability of PPIs, with contributions between various hot regions can be either additive (29) or cooperative (30). Hotspots are also defined as residues that impede protein–protein interactions if mutated (31). Hotspots tend to occur in clusters and are in contact with each other resulting in hot regions, a network of conserved interactions (32). Antibodies are considered widely acceptable approach to target PPIs, since they could target or bind a vast region covering the discontinuos tertiary epitopes. Hence, while designing small molecule agents, the compounds need to be targeted to interact with either in one hot region (either hydrophobic or hydrophilic), which could be a critical contributor to the signaling pathways or need to engage with critical residues in multiple hot regions (both hydrophobic and hydrophilic) if an additive effect on hotspots is required for the desired pharmacological effects. In agreement with the concepts described above, the identification of small molecule PPI inhibitors have been possible because of the presence of PPI hotspots (33, 34).

Advances in solving the crystal structure of PD-1:PD-L1 and PD-1:PD-L2 complexes and mapping of the molecular networks resulted in the identification of several potential hotspots. Three major hot regions identified on PD‐L1 based on PD-1:PD-L1 crystal structure are (1) hydrophobic pocket consisting of the side chains of Tyr56, Glu58, Arg113, Met115, and Tyr123; (2) second pocket nearby hydrophobic cleft composed of Met115, Ala121, and Tyr123; and (3) extended groove made up of the main chain and side chains of Asp122, Tyr123, Lys124, and Arg125 (35). In the PD-1:PD-L1 interface, hot region containing hydrophobic regions are involved in hotspots 1 and 2 and are proposed to be ideal for binding to PD-L1 using conventional small molecules. Hotspots in the PD1:PD-L interaction have also been quantitatively predicted based on the theoretical calculations (36). This analysis has identified six hot spots on PD-L1 (Tyr123, Tyr56, Arg125, Met115, Arg113, and Gln66) and two warm spots on PD-L1 (Ile54 and Lys124) (36). Among various hotspots/hot regions reported, the hydrophilic hot region containing polar residues is reported to be a solvent exposed with extended shallow groove and with multiple hydrogen bond donors and acceptors and is considered to be challenging to target *via* conventional small molecules abiding the famous rule of five (37).

## Two Major Classes of Small Molecules Targeting PD1-PD-L1 Axis

A survey of the literature as highlighted in this section below indicates predominantly two different classes of small molecule inhibitors targeting PD-L1, namely, (a) compounds based on the biphenyl scaffold, originally identified by a conventional approach of screening compounds in a binding assay and (b) amino acids-inspired small molecules mimicking the receptor–ligand interface identified in a functional assay.

### Biphenyl Derivatives

**Figure 1:**
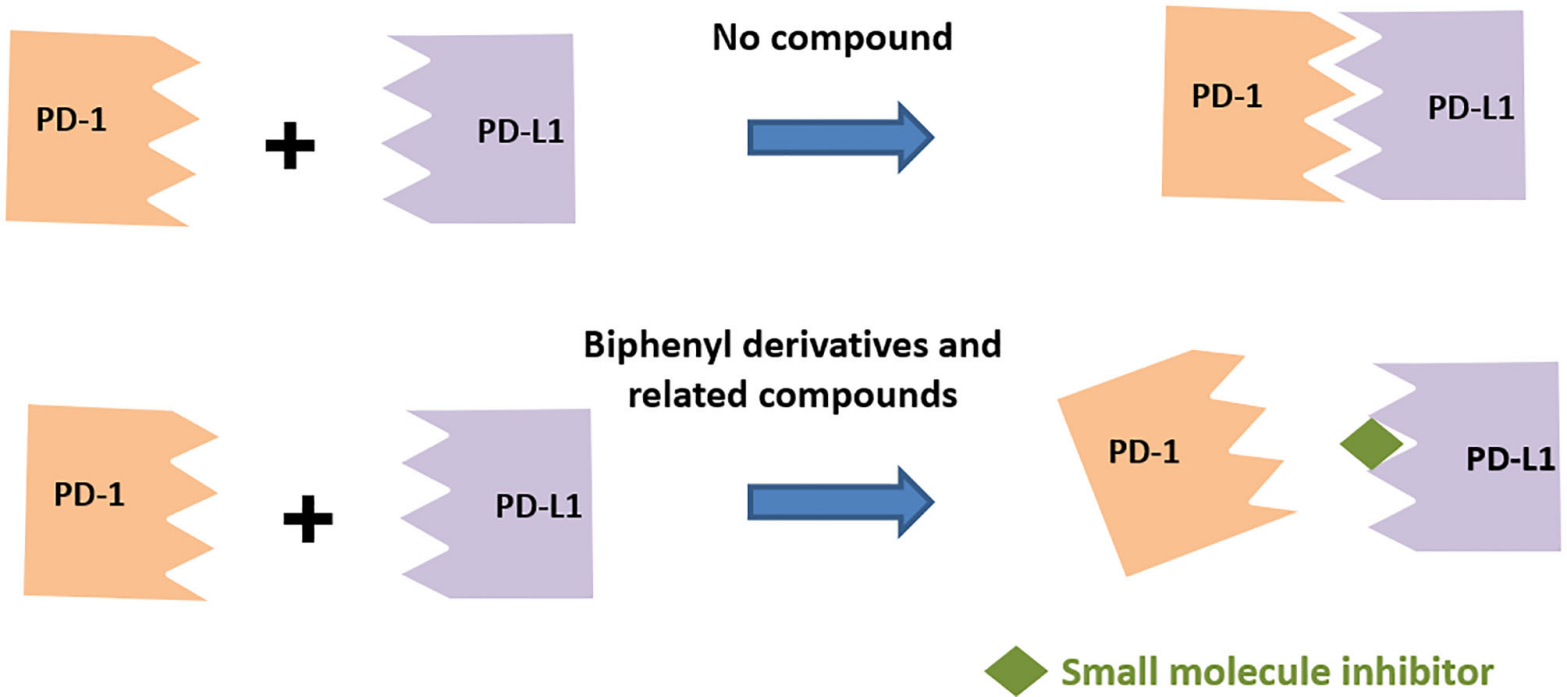
Compounds based on the biphenyl scaffold, originally identified by a conventional approach of screening compounds in an assay for prevention of the PD-1, PD-L1 interaction.

**Figure 2:**
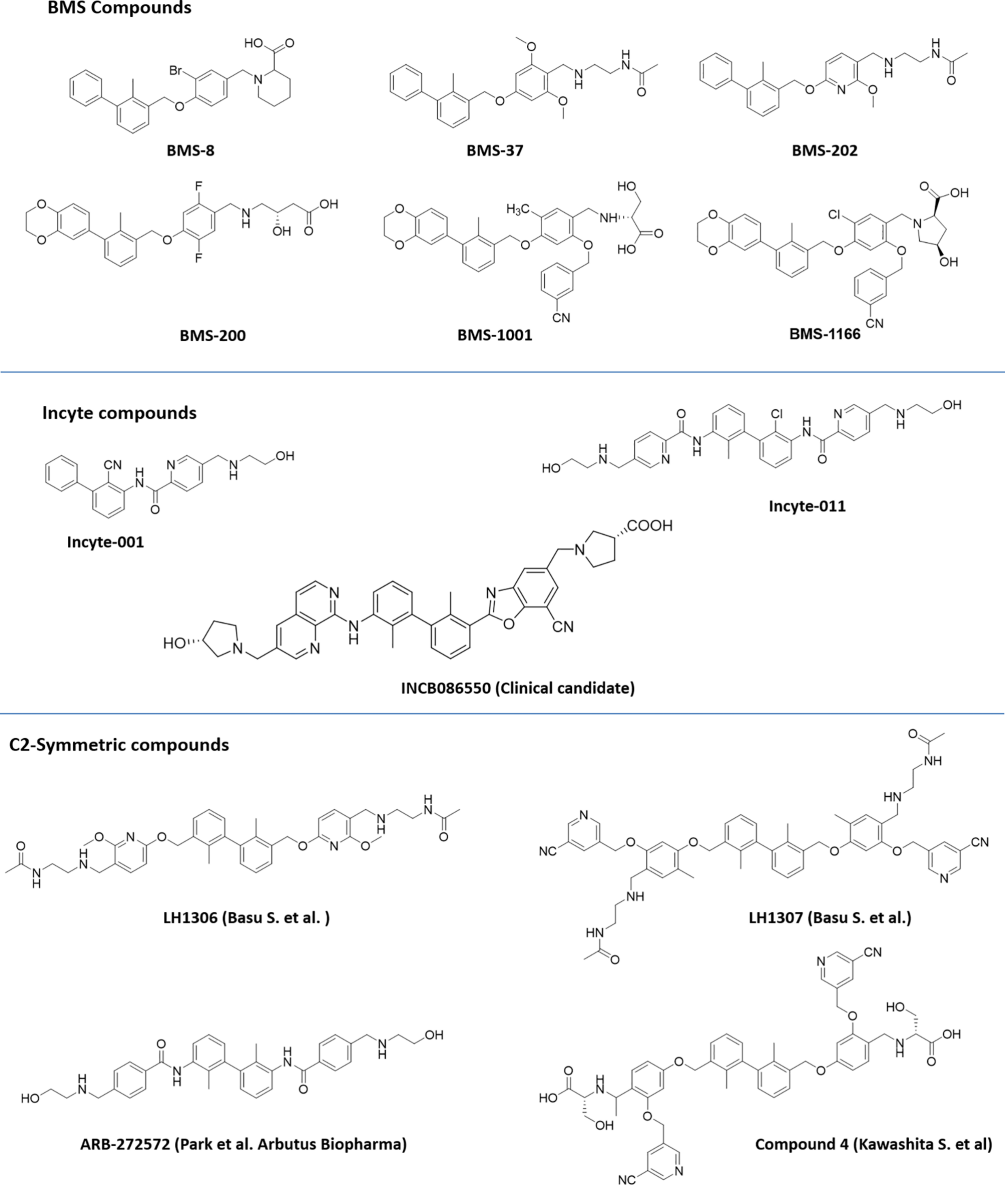
Representative structures of first-generation biphenyl derivatives and C_2_ symmetric compounds targeting PD-L1.

Scientists from BMS reported a series of substituted biphenyl derivatives based on their ability in preventing the PD-1:PD-L1 interaction. Extensive characterization and elucidation of the mode of action of these compounds (**Figure 1**) have been reported in several publications (discussed in detail in the following section). However, further advancement of these interesting but highly hydrophobic small molecules into the clinic has not been reported. The contributions of hotspots for stabilizing PPIs can be either additive or cooperative, and if the targeting of one of the hotspots is not good enough to achieve the desirable activity, design strategies need to be included to cover one or more hotspots. Based on this rationale, the binding mode of Compound 2a (BMS-1001 in **Figure 2**), which contains the 2,3-dihydro-1,4-benzodioxinyl group and polar groups, is more effective in dimerization of PD-L1 ligands with a network of hydrophobic and polar interactions compared to the first-generation BMS-type compounds, which can bind to only hydrophobic cleft (38). Since the last 6 years, several companies including Incyte Corporation, Arising International Inc., Chemocentryx Inc., Polaris Pharmaceuticals, and Guangzhou Maxinovel Pharmaceuticals Co. have discovered a series of small molecule PD-L1 inhibitors based on the biphenyl core (39–41).

Among the new modified biphenyl scaffolds, compounds with C_2_ symmetry or pseudosymmetry containing polar groups are gaining a lot of attention than asymmetric structure (**Figure 2**). The C_2_−symmetric small molecule inhibitors of PD-1:PD-L1 interaction containing the lipophilic biphenyl core for binding to PD-L1 at its PD-1 binding site to occupy the hydrophobic cleft have been reported (42). NMR and X-ray co-crystal structural studies indicated that symmetric molecules such as LH1306 and LH1307 induce formation of a more symmetrically arranged PD-L1 dimer than previously reported asymmetric inhibitors. The polar groups 2-(acetamido) ethylamine of BMS-202, incorporated into the C_2_ symmetric molecules, LH1306 and LH1307, are reported to extend out the hydrophobic cleft and occupy the solvent-exposed region sandwiched between the AG and C′C β strands of the respective PD-L1 proteins, further enhancing binding to the two PD-L1 monomers *via* hydrogen bonds and electrostatic interactions. It was also hypothesized that a symmetric compound would induce a flip of sidechain of Tyr56 protein residue to form a new cavity, leading to increased binding affinity to PD-L1, and PD-1/PD-L1 inhibitory activity in physiological conditions as reported for Compound 4 (43). The improved cellular activity of the PD-L1 inhibitor ARB-272572 further demonstrated the importance of C_2_-biphenyl skeleton and symmetry with polar (2-hydroxyl ethyl)amino group extended from the hydrophobic cleft (44). Representative structures of compounds disclosed by BMS, Incyte, and C_2_ symmetric compounds are presented in **Figure 2**.

**Figure 3:**
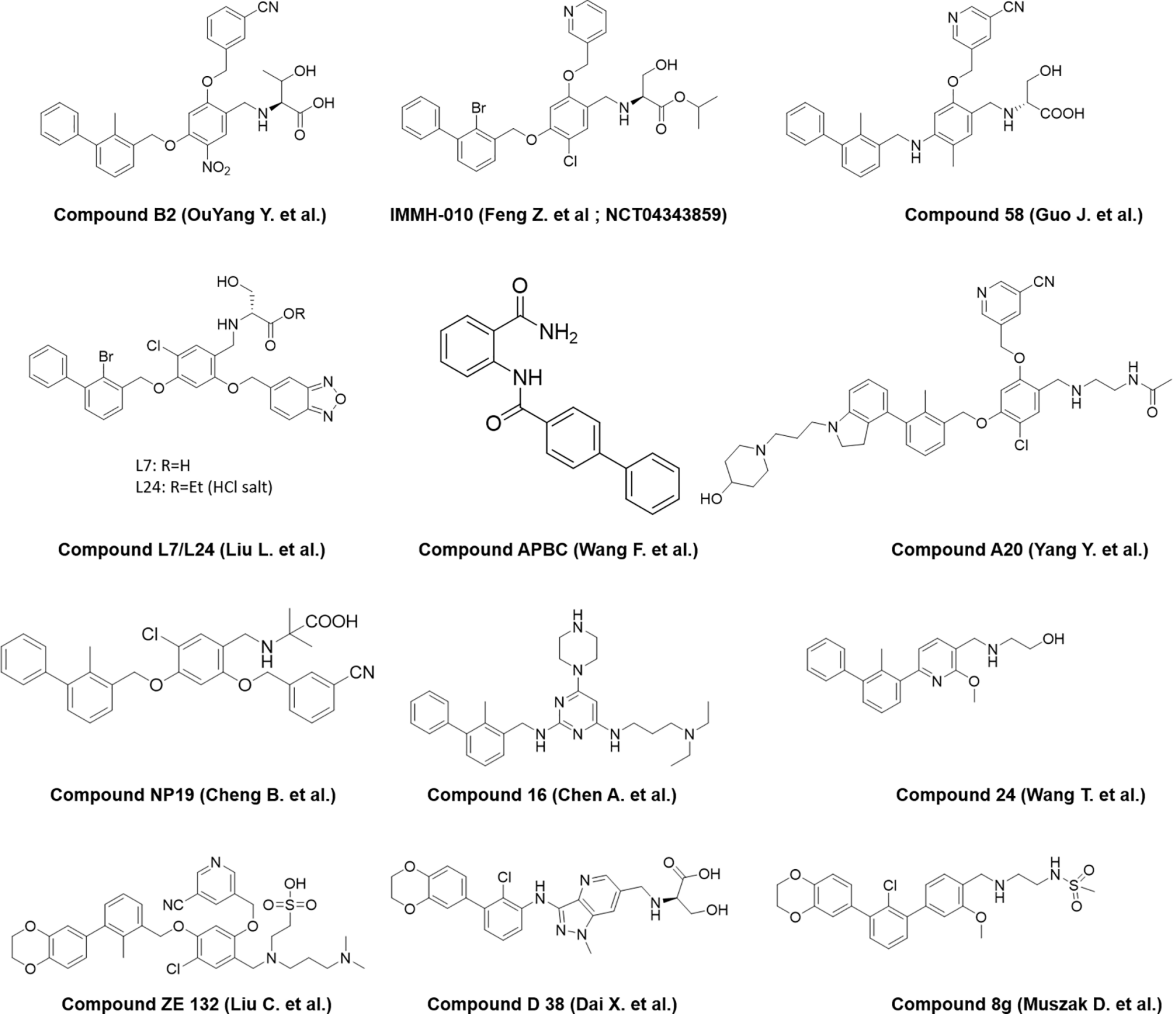
Representative structures of modified biphenyl analogues without C_2_ symmetry but with improved druggability.

A number of reports are also emerging recently from the industry and academia using the privileged structure of biphenyl-containing compounds and their various derivatives to improvise the druggability of the molecules. This includes scaffold based on nicotinyl alcohol ether derivative (45–47), resorcinol dibenzyl ether (48), 4-phenylindoline derivatives (49), combining two privileged scaffolds such as biphenyl backbone structure and 2-amino-pyrimidine structure (50), biphenyl-4-carboxamide derivatives (51), incorporating taurine moieties (52), 1-methyl-1H-pyrazolo[4,3-b] pyridine derivatives (53), replacing the linker connecting aryl group and biaryl core with a novel amine linker (54), a series of novel biphenyl pyridines derivatives lacking linker (55), biphenyl methyl nitrophenyl core unit (56), and terphenyl scaffold derived from the rigidified biphenyl inspired structure (57). Representative structures of the compounds disclosed are presented in **Figure 3**.

### Amino Acid Inspired Interface Mimics

The presence of distinct interface of PPIs involving the loops/strand sequences can be exploited in identifying peptides that mimic the native interaction interface. Checkpoint proteins are membrane proteins with majority of them from the B7 family (58). Most of the members of the B7 family and their ligands belong to the immunoglobulin superfamily (IgSF). All IgSF proteins have a characteristic Ig-fold structure with an IgG domain consisting of anti-parallel β-strands and loops connecting the β-strands with most strands constituting ~10 amino acids. Receptor–ligand interaction of IgSF proteins is mediated either through loops, strands, or loops and strands. Rational design of peptides based on these interacting interfaces is a proven strategy for design of PPI inhibitors (59). However, due to the inherent limitations of peptides in general, the focus has now shifted to peptide mimics by depeptidizing the short stretch of residues with cyclic linkages, un-natural linkages, retro and retro-inverso amides, urea, and non-amide bonds as peptide bond surrogates.

Proteins are built from a set of only 20 amino acids, and the side chains, which are unique to each amino acid, exhibit different chemistries. The side chains of amino acids involved in hotspots residues are crucial and participate either in polar contacts or hydrophobic interactions in the interface of PPI contributing to stability and specificity of the interaction. In general, a peptidomimetic is referred as bioactive peptide mimicking molecules with the side chain groups of critical amino acids oriented to enable bioactive conformation to yield desirable pharmacological properties (60). The peptidomimetic structure ranges from molecular scaffolds replacing the peptide backbone, to compounds with modified peptide sequences with improved therapeutic properties (61–64).

**Figure 4:**
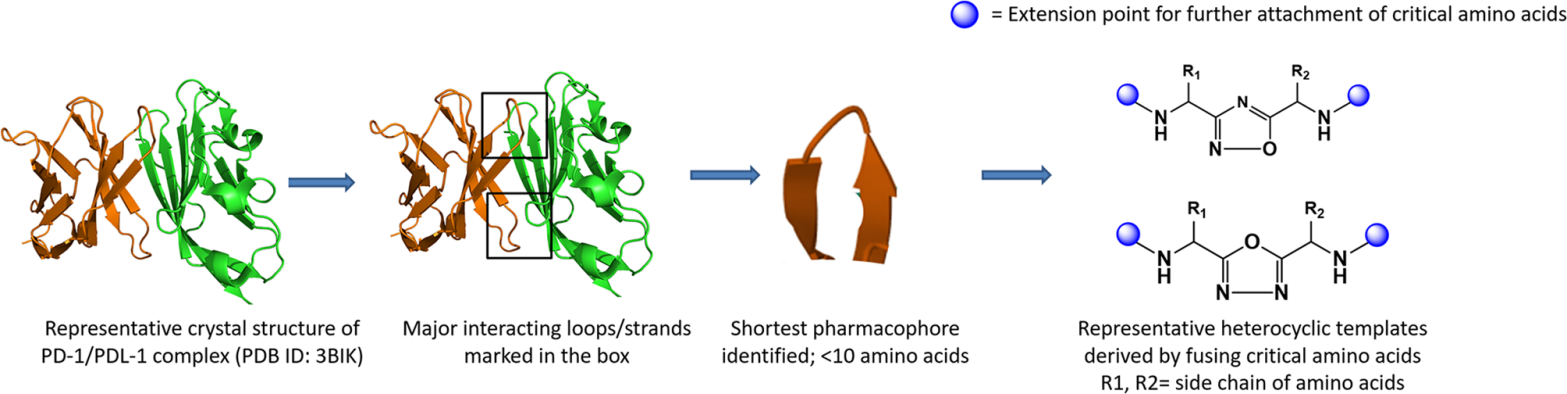
Amino-acid-inspired small molecules mimicking the receptor–ligand interface identified in a functional assay.

**Figure 5:**
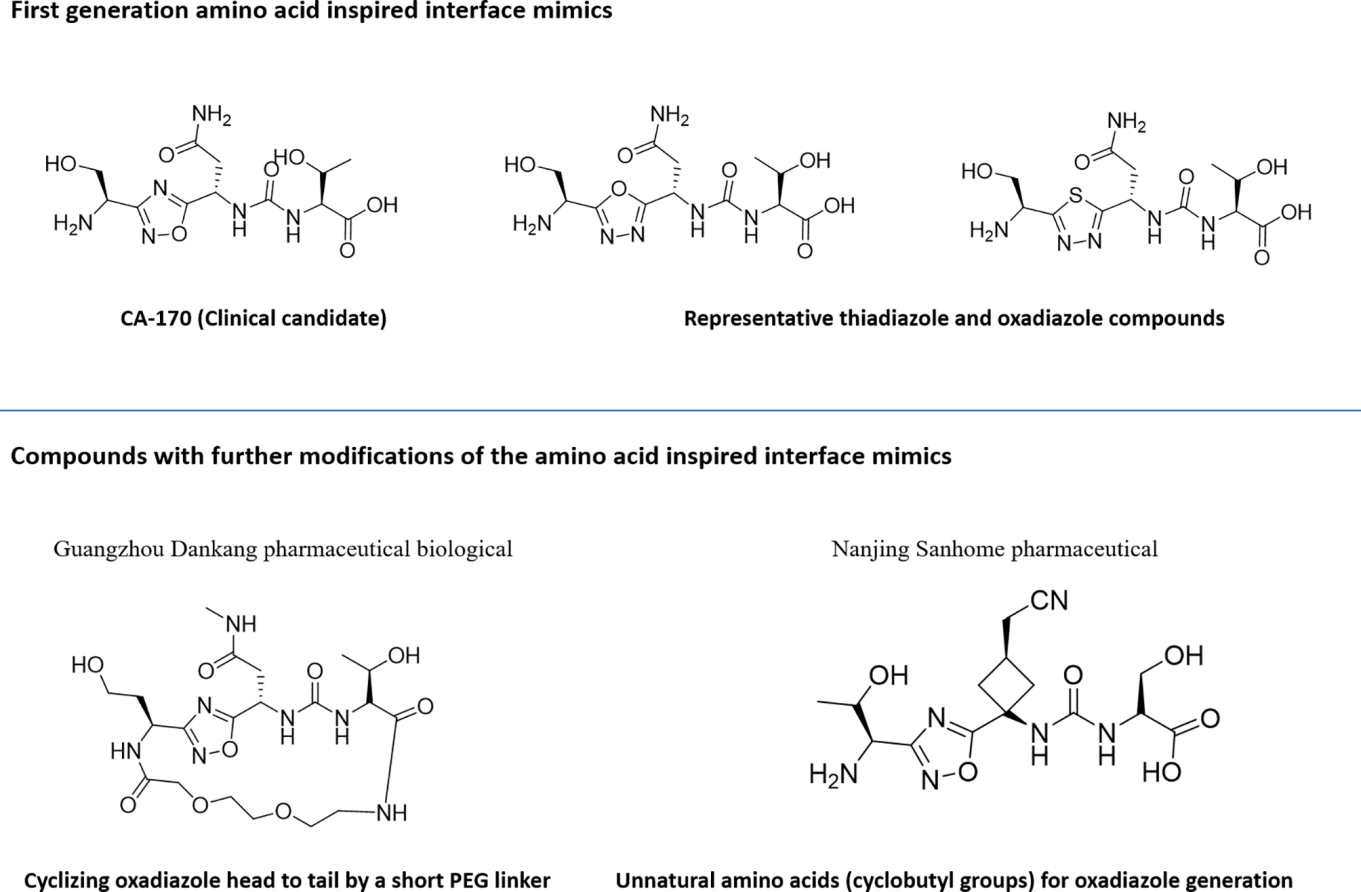
Representative structures of amino-acid-inspired interface mimics targeting PD-L1.

Utilization of the design principles described above, several peptidomimetics were identified, but none of the peptidomimetics capable of antagonizing the PD-1 signaling exhibit oral bioavailability (65). Therefore, the native interaction pharmacophore first identified by the reductionist approach comprising of three to five amino acids, as in the case of typical peptidomimetic, was radically redesigned (**Figure 4**). In this design strategy, compounds with side chain functionalities of the amino acids from the native interaction pharmacophore protrude from a heterocyclic template with no amide bonds, in which the side chains are the critical interacting motifs of PPI hotspots, while the metabolically stable heterocyclic template anchors the amino acid side chains presumably in a required geometry (**Figure 5**). The novel heterocyclic oxadiazole templates were obtained by fusing two amino acids while presenting the desired amino acid side chains to interact with residues on PD-L1, while bringing in the remaining amino acid(s) from the pharmacophore through the use of linkers such as urea bonds resulting in the compound such as CA-170 (19, 66–68).

Recently, scientists from Guangzhou Medicine Pharmaceutical Biological Co. Ltd. and Nanjing Sanhome Pharmaceutical disclosed in their patent applications the close analogues of the lead PD-L1 inhibitor compound from Aurigene approach: (a) cyclizing oxadiazole head to tail by a short PEG linker (69) and (b) incorporating unnatural amino acids majorly containing cyclobutyl groups for oxadiazole generation instead of α-amino acid reported by Aurigene (70). The detailed profile of these compounds is yet to be published.

As discussed earlier in this article, to improve the clinical response rate, one of the strategies would be to target simultaneously more than one non-redundant checkpoint pathways. It was hypothesized that a substantially reduced native pharmacophore derived from protein–protein interacting interfaces of one of the B7 family protein (such as PD-1) also has the potential to interact with other proteins belonging to IgSF (such as VISTA and TIM3) with structurally similar grooves induced by pockets of sequence similarity (71). A focused library was designed and synthesized using amino acids in the hotspot region including conserved residues in the hotspot regions to identify selective or spectrum-selective inhibitors targeting one or more non-redundant checkpoint signaling pathways such as PD-L1 and VISTA (72), PD-L1 and TIM3 (73, 74), VISTA (75), TIM-3 (76), PD-L1 and T-cell immunoreceptor with immunoglobulin and ITIM domain (TIGIT) (77), TIGIT (78), and cluster of differentiation 47 (CD-47) (79) pathways with desirable physicochemical properties and exposure upon oral administration. It is worth noting that small molecules with dual immune checkpoint inhibition have only been reported from the amino-acids-inspired interface mimic approach.

## Characterization of Binding to PD-L1 and Prevention of PD1:PD-L1 Complex Formation

Most reported small molecule inhibitors of PD-L1 have been extensively characterized for binding to PD-L1 in various biophysical and biochemical assays using extracellular domain of PD-L1 from heterologous expression system. Scientists from BMS were the first to report biphenyl compounds identified using a homogenous time-resolved fluorescence (HTRF)-based binding assay in order to screen and rank their ability to inhibit PD-1:PD-L1 complex formation (80). Based on BMS scaffold, several molecules up to sub-nanomolar binding potency in HTRF assays have now been reported both from the industry and academia (39, 41, 79, 81). Direct binding of biphenyl compounds such as BMS compound 37 to PD-L1 has been demonstrated *via* SPR and ELISA-based assays (82). Using such biophysical techniques, it was observed that the amino-acids-inspired interface mimic CA-170 did not show observable binding to either PD-L1/PD-1 or inhibition of the PD-1:PD-L1 interaction while acknowledging the key differences in the “expressed” proteins and real-life interacting proteins as expressed on the surface of T cells and APCs (82).

The binding of biaryl scaffold-based compounds from BMS using three independent binding assays was demonstrated using DSF, MST, and SPR techniques and the concentration-dependent inhibitory activity in the PD-1/PD-L1-based biochemical ELISA (83). A higher affinity for BMS macrocycle peptide, which contains hydrophobic amino acids as compared to BMS biaryl scaffold compounds, was reported in DSF. However, the results of high affinity for macrocycles in DSF assay are interpreted with a caution due to the different physicochemical properties of macrocyclic peptide. In a similar assay, the binding affinity of BMS compounds has also been established by MST assays using fluorescent-labeled PD-L1 protein instead of label-free PD-L1 protein as used for DSF for incubation with test compounds.

The recent understanding of C_2_ symmetric compounds targeting both hydrophobic cleft and polar groups in PD-L1 revealed that symmetric compounds have a better binding in HTRF assays as compared to asymmetric compounds. The C_2_-symmetric inhibitor LH1306 (IC_50_ = 25 nM) was reported to be 3.8-fold more potent than its asymmetric counterpart (42). In this study, authors also illustrated the importance of 1,1′-1,1′-biphenyl core by comparing the core generated with 1,4- or 1,3-phenylene and 1,4- or 2,6-naphthalenylene analogues (micromolar binding activity). The importance of symmetric architecture for PD-L1 inhibitors was demonstrated with the compound, ARB 272542 (44), which inhibited PD-1/PD-L1 in a cell-free HTRF at 400 pM IC_50_ compared to 200 pM (anti-PD-L1 antibody, MIH1) and 200 pM (anti-PD-1 antibody, nivolumab). Incyte compounds with asymmetric and symmetric structure were analyzed by Liu et al., and data further confirmed that C_2_ symmetric compound (Incyte 011, 5.3 nM) was almost twice as potent as its asymmetric form (11 nM) (84).

NMR has been extensively used to characterize the binding of reported PD-L1 inhibitors to PD-L1. Prof. Holak and colleagues initially reported SAR-by-NMR approach to demonstrate the direct binding of BMS compounds to PD-L1. In this method, ^15^N-labeled PD-L1 was titrated with increasing amount of tested compound, and signals were monitored using 2D ^1^H-^15^N heteronuclear multiple quantum coherence (HMQC) NMR experiment. Significant shifts in the correlation NMR signals of PD-L1 upon addition of each tested compound documented their direct binding to PD-L1 (85). The ability of BMS-202 to disrupt PD-1/PD-L1 interaction was further demonstrated in a new antagonist-induced dissociation assay NMR screen, called AIDA-NMR (85, 86). In this method, ^15^N labeled PD-1 was initially titrated with slight excess of non-labeled PD-L1 so that no further changes in the linewidth of the 1^H^-^15^N resonance peaks was observed as monitored by HMQC. The addition of test compounds inhibiting interaction or dissociation of this preformed complex results in the narrowing of ^1^H-^15^N signals. The NMR competition assay was further improvised, called weak-AIDA, for the fragment screening of weak binders using a low-affinity mutant of PD-1 (87). The weak-AIDA assay was conceptualized by introducing point mutations in the complex’s protein that is not targeted by the inhibitor resulting in a low-affinity complex, thus allowing for short fragments to dissociate the complex. The method is illustrated using the compounds that block the Mdm2/X-p53 and PD-1/PD-L1 interactions (87). Among the various mutations attempted, Asn66 to Ala mutation was considered as the ideal mutation to generate mutant PD-1 protein due to its thermal stability as compared to native PD-1, retaining the overall structure and minimum interaction with the binding partner. This methodology was further utilized to determine the minimal fragment of BMS-1166 responsible for the PD-L1 binding (88). Although protein has been mutated at the asparagine position distant from the interacting site, considering the importance of asparagine residues in post-translational modifications, these variations may need to be checked with its impact in physiological environment (89). PD-1 is heavily glycosylated at Asn49, Asn 58, Asn 74, and Asn 116 in T cells, and Asn 58 is highly critical for PD-1/PD-L1 interaction (89) underscoring the need for structure analysis using the glycosylated protein. Given that glycosylation of PD-L1 (90) is also critical for binding to PD-1, it would be important to determine the structure of PD-1/PD-L1 binding using the glycosylated forms of these proteins in the future to better understand the interaction between PD-1 and PD-L1. The criticality of using protein with all appropriate post-translational modifications for interaction with reported ligands, especially those identified in cell-based assays, has been elegantly shown for the binding of a small molecule to p53 (91, 92).

Published findings utilizing solution NMR (93) and other biophysical techniques (83) have indicated a lack of binding of the amino-acids-inspired interface mimic, CA-170 to PD-L1. The lack of conclusive binding in these studies may be due to the use of protein expressed in *E. coli* deficient in appropriate post-translational modifications and devoid of functional activity. To recapitulate the interaction as in the native environment, a cellular NMR spectroscopy using PD-L1-expressed mammalian cells in the native context along with the parental cell line as a negative control was utilized (19). Cell-based NMR spectroscopy is an ideal tool for studying the binding of macromolecules with a ligand under the physiological environment to obtain biologically relevant structural and functional information (94, 95). This is achieved by monitoring the difference in the nuclear-spin relaxation or transverse relaxation (T_2_) rates of a small molecule ligand in the presence and the absence of the macromolecular targets such as cells (96). There is a huge difference in the rotational and translational motion of small molecule ligands and large molecule receptors. Small, rapidly tumbling molecules have much slower relaxation rates than slowly reorienting cells, and therefore, the T_2_ of small molecules is generally large compared to the T_2_ of large macromolecules or cells. This phenomenon is exploited to detect and characterize binding by measuring the T_2_ values of the ligands in the absence and presence of the macromolecular targets like proteins or cells (96, 97). The ligands associated with macromolecules or cells attain the NMR properties of macromolecules or cells. This is analogous to imagining a small molecule agent as an ant and a cell as an elephant and the ant sitting on top of the elephant moving with a speed of the elephant. Based on the different relaxation rates of small molecules and cells, the interaction of CA-170 to PD-L1 was confirmed in the cellular context using PD-L1 overexpressed CHO-K1 cells in comparison to blank CHO-K1 cells (19). The results from these studies demonstrate that the cellular NMR technique is sensitive in determining the complex binding mechanism in a physiologically relevant environment.

## Characterization of Binding to PD-L2

Interestingly, despite a flurry of publications in the recent past, limited information is available regarding the interaction of small molecule agents reported to bind to PD-L1 for their binding to PD-L2. In one of the early publications, BMS compounds were evaluated for their interaction with PD-L2 (85). Data indicated their selective binding to PD-L1 and not to PD-L2. In view of the high degree of homology between PD-L1 and PD-L2, it would be of great interest to characterize newer biphenyl compounds including symmetric compounds with greater potency for their interaction with PD-L2.

## Analysis of Functionality

In a simplistic view, because of the presence of domains of PD-L1 interacting with the small molecule agents outside of the cells, a good correlation for potency between binding leading to inhibition of PD-1:PD-L1 complex formation and functional assays is expected. However, it is worth noting that not all molecules with demonstrated binding to PD-L1 and/or capable of preventing PD-1:PD-L1 complex with high affinity have shown good activity in functional assays. Various assays utilized for functional characterization include measurement of impact on signaling components, e.g., the downstream transcription factor such as NFAT, using a luciferase-based reporter assay or signaling intermediate SHP-1 and primary T-cell-based assays in which proliferation or cytokine secretion is monitored in a set-up involving antigen recall or rescue from the inhibitory effect of PD-L1. Details of the most commonly used functional assays are summarized in **Table 2**.

**Table 2:** Most commonly used functional assays to monitor the impact of small molecule agents on PD-1 pathway.

Type of assay	Source for PD-1	Source for PD-L1	Consequence of interfering in the PD-1:PD-L1 interaction by small molecules	Detection system	Comments
NFAT reporter	Jurkat (immortalized line of human T cells) overexpressing PD-1 and luciferase gene controlled by the NFAT-response element	CHO-K1 (Chinese hamster ovary cells) cell line overexpressing PD-L1 and T-cell receptor activator	Activation of TCR signaling leading to greater luciferase expression	Chemiluminescence	Engineered cell line as T cells in the assay. Assay commercially available
SHP-1	Jurkat cells expressing PD-1 and SHP-1 each fused with different individually inactive fragments of the β-galactosidase	U-2 OS osteosarcoma cell line expressing PD-L1	Decrease in SHP-1 recruitment leading to lower β-galactosidase activity due to reduced enzyme fragment complementation	Chemiluminescence	Engineered cell line as T cells in the assay. Assay commercially available
PBMC	T cells activated by anti-CD3/anti-CD28	Recombinant protein	Activation of TCR signaling leading to the rescue of proliferation or cytokine release	FACS or ELISA	Primary T cells in the assay—closer to physiological context
PBMC/whole blood	T cells activated by staphylococcal enterotoxin B (SEB) or CMV antigen	Other cells in PBMCs or blood; not controlled likely leading to greater variability in the assay	Activation of TCR signaling leading to the rescue of proliferation or cytokine release	FACS or ELISA	Primary T cells in the assay—closer to physiological context

### NFAT Reporter Assay

In most of the PD-L1 inhibitor publications, a co-culture assay in which CHO-K1 cells (of Chinese Hamster ovary origin) overexpressing PD-L1 and T-cell receptor activator with the effector Jurkat T cells overexpressing PD-1 and a luciferase gene controlled by the NFAT response element has been used. In such a reporter assay, BMS-1001 and BMS-1166 restored the activation of Jurkat T cells, represented by an increase in luciferase activity (98). However, the potential of the compounds in restoring the activation of effector cells is significantly lower than that observed for the therapeutic antibodies and with lower maximal cell activation levels. Such drastic reduction in the potency of BMS compounds (83) and other derivatives based on the original BMS compounds (49) in the reporter assay has also been confirmed by several other groups (42, 44, 53, 57, 99). In a similar reporter assay system, CA-170, which rescues T cells from the inhibitory activity of PD-L1, did not show activity (93), while a low activity was observed (<1.5 induction of reporter activity at doses ranging from 0.1 to 10 µM) with the most potent small molecule capable (BMS-1166) of potently disrupting the interaction of PD-1:PD-L1 complex (IC_50_ 7 nM in TR-FRET assay). In comparison, a significant activity was observed for macrocyclic peptide or nivolumab in the same study. Another biphenyl compound, L7, has also been reported to show significantly lower potency compared to its activity in HTRF-based biochemical assay for the disruption of PD-1-PD-L1 interaction (99). Interestingly, the more potent two C_2_-symmetric inhibitors LH1306 and LH1307, with IC_50_ value of 25 and 3 nM, respectively, dose dependently released the PD-1-mediated inhibition of PD-1-expressing Jurkat T cells and induced the activity of luciferase with EC_50_ values of 4.2 and 0.76 µM, respectively, and the asymmetric version of LH1306 was inactive (42). Similarly, ARB-272572, another symmetric molecule with potent activity (400 pM IC_50_) in PD-1/PD-L1 HTRF showed significantly lower potency (>40-fold less in the reporter assays, whereas anti-PD-L1 and anti-PD-1 antibodies showed comparable potency in binding and reporter assays (44). Taken together, the available data suggest that the reporter assay system, which is far removed from the physiological context, may not be ideally suited for characterizing all classes of small molecule inhibitors.

### SHP-1 Signaling Assay

Another heterologous functional assay based on the enzyme fragment complementation technology, the commercial PathHunter cell-based PD-1 (SHP-1) signaling assay, has also been used to investigate the potency of PD-L1 inhibitors. This assay utilizes a stable, engineered cell line with enzyme fragment complementation technology for use in studying drug candidates targeting PD-1/PD-L1 pathway. In this assay format, symmetric compounds LH1306 and LH1307 demonstrated 8.2-fold (EC_50_, 334 nM) and 2.8-fold (EC_50_, 79 nM) more activity as compared to their asymmetric analogues in commercial PathHunter cell-based PD-1 (SHP-1) signaling assay underscoring the need for C_2_-symmetry for optimal functionality (42). However, it is important to note that as in the NFAT reporter assay, the potency of the compounds in the SHP-1 signaling assay was ~10-fold lower compared their potency in PD-1:PD-L1 disruption.

### Primary T-Cell-Based Assay

Only a limited number of publications report the characterization of biphenyl compounds in the primary T-cell-based assays. In a peripheral blood mononuclear cell (PBMC)-based assay, BMS-202, one of the early biphenyl compounds with poor cellular activity in the NFAT reporter assay (53), showed significant rescue of interferon gamma (IFN-γ) secretion inhibited by PD-L1 at a lower concentration (100 nM) similar to another biphenyl compound, Compound 58 (54). The biphenyl compound B2 has been shown to induce IFN-γ secretion from the lymph node T cells co-cultured with LLC cells and rescue the inhibition of IFN-γ secretion from PD-L1 of PBMC treated with anti-CD3/anti-CD28, with a potency similar to that observed in biochemical assay for the disruption of PD-1/PD-L1 interaction (56). During the discovery of amino-acid-inspired interface mimic CA-170, a PBMC-based function assay monitoring the rescue of T cells from the inhibitory activity of PD-L1 in inducing proliferation and cytokine secretion was extensively used. In this assay, CA-170 shows the Emax and potency similar to those observed with an anti-PD1 antibody (19). A functional assay in a similar format was recently employed to establish equipotent functional activity as in biochemical assays of modified biphenyl compound, Compound B2, from researchers of Nanjing University and China Pharmaceutical University (56).

It is important to note that not all studies have reported a good correlation between biochemical and PBMC-based functional assays. For example, Compound 24, a biphenyl asymmetric molecule, showed about 30-fold lower activity in PBMC-based tumor cell line (MDA-MB231) killing assay (Wang 2021). In a co-culture assay using 293T-OS8-hPDL1 cells, and CD3 + T cells, Incyte-011, a symmetric compound, increased IFN-γ production in a dose-dependent manner and reached the peak at the maximum dose of 1 μM, indicating a significant attenuation relative to its biochemical activity (IC_50_ of 5 nM in HTRF assay), whereas the asymmetric compound Incyte-001 and BMS compounds were not active in this assay. In the same assay, the control PD-L1 antibody atezolizumab was highly active, with IFN-γ production reaching up to 350 pg/ml at 5 nM concentration (84).

Based on the observation that PD-1 antibodies have been shown to upregulate T-cell responses in response to superantigens such as staphylococcal enterotoxin B (SEB) or cytomegalovirus (CMV) antigens, these assays have also been utilized for the characterization of PD-L1 agents. In the SEB-induced PBMC assays, to monitor proliferation and secretion of IL-2 or IFN-γ production, BMS small molecule compounds showed activity only at micromolar concentration, while BMS macrocyclic peptide induced high levels of IL-2 at sub-micromolar concentrations (83). In a separate study, a symmetric compound, ARB-272572, that demonstrated activity in an NAFT reporter assay of 17 nM IC_50_ displayed a potency of 3 nM in CMV antigen recall assay, still a significant drop-off in the potency relative to that observed in biochemical activity (400 pM IC_50_ in a cell-free HTRF assay) (44).

Among all the functional assays employed, the best correlation with biochemical potency was observed for selected biphenyl compounds using primary T cells with PBMCs as the source and in the presence of PD-L1. Interestingly, CA-170, which failed to exhibit activity in HTRF based PD-1:PD-L1 disruption and NFAT-reporter-based functional assay, was highly active in the PBMC-based assays, which has now translated into immune activation observed in the clinic (100). Taken together, these findings raise the possibility of PBMC/primary T-cell-based assays, which are used to monitor rescue from the inhibitory activity of PD-L1, in which the addition of PD-L1 brings in the specificity, as the most relevant assay to analyze the immune activation potential of small molecule PD-L1 agents. While this possibility needs to be validated further, it may not be entirely surprising considering that PD-1:PD-L1 interaction is studied in a setup that is a lot closer to the physiological and therapeutic context. It is also important to note that in the PBMC-based assay format, the substitution of human PBMCs with monkey PBMCs or mouse splenocytes along with PD-L1 of monkey or mouse origin allows to evaluate the cross-species functional activity (19).

In the cellular pharmacology studies using human T cells and the rescue of inhibition by PD-L1, both CA-170 and anti-PD-1 antibodies showed a bell-shaped T-cell activation response (101). In these assays, optimal T-cell activation occurs between concentrations of 125–500 nM beyond which there is a substantial reduction in the activity. Such inverse dose response has also been observed *in vivo* in tumor models with a series of novel benzo[c][1,2,5]oxadiazole derivatives with potent PD-L1 binding and functional activity (99). An inverse dose response was observed with PD-L1-humanized MC38 model, in which compound L24 exhibited significant antitumor efficacy in this model, with 25 mg/kg (TGI 44.2%) exhibiting higher efficacy compared to a higher dose of 50 mg/kg (TGI 30.8%). The likely cause for such a bell-shaped response is the hyperactivation of the immune system leading to activation-induced T-cell death (102), which is detrimental to the effect of the drug.

### Functional Antagonism of PD-L2

Among the reported small molecule agents binding and interfering in the function of PD-L1, CA-170 has also been shown to functionally antagonize signalling from PD-L2 (19). In human PBMC or mouse splenocyte-based assays, CA-170 has shown dose-dependent rescue of inhibition of proliferation or cytokine secretion in the presence of either PD-L1 or PD-L2. The potency of CA-170 in these assays for functional antagonisms were similar to that observed with commercially available anti-PD1 antibodies (19), which are known to antagonize signaling originated from either PD-L1 and PD-L2. Unlike PD-L1, the expression of PD-L2 is mainly restricted to professional antigen-presenting cells (APCs) like macrophages and dendritic cells (DCs) and more commonly upregulated in lymphoid malignancies (103). Studies have also indicated that the expression of PD-L2 is independently associated with clinical response in pembrolizumab (anti-PD1)-treated patients, supporting that the presence or absence of PD-L2 expression may also play a role in response to PD-1 axis targeted therapies (104). The consequence of selective inhibition of PD-L1 versus dual inhibition of both PD-L1 and PD-L2 needs to be characterized, specifically in view of greater apparent clinical efficacy of anti-PD1 antibodies capable of blocking signals that originated both from PD-L1 and PD-L relative to the efficacy from anti-PD-L1 antibodies.

## Mechanism Of Action of the Reported Small Molecule Compounds Targeting PD-L1

Even though the functional antagonism of the PD-1 pathway is anticipated from a compound capable of binding to one of the partner in the PD-1:PD-L1 complex and preventing the interaction, emerging data point to additional complexity with which reported compounds antagonize the PD-1 signaling.

### Induction of PD-L1 Dimerization and Inhibiting PD-1:PD-L1 Interaction

The detailed characterization of PD-L1 targeting compounds originally described by the BMS group was carried out by Prof. Holak and colleagues, and they have extensively published on the mechanism of action, binding mode based on X-ray structure determination, confirmation of binding, and functionality by various methods (35). One of their major contributions to the field is in identifying the molecular details of the human PD-1/PD-L1 interaction using X-ray structure of hPD-1:PD-L1 complex, which led to the identification of three hotspot pockets in PD-L1, the binding of which can lead to inhibition of PD-1:PD-L1 interaction (35). Among these pockets, two are rich in hydrophobic amino acids with considerable hydrophobicity and one in hydrophilic amino acids. The direct binding of BMS compounds to PD-L1 *via* NMR assays and the structural basis for these compounds (BMS-8 and BMS-202) in targeting PD-L1 were delineated (85). Using crystal structure studies, it was demonstrated that these compounds inhibit the PD-1:PD-L1 interaction by inducing PD-L1 dimerization through PD-1 interacting surface. The BMS compounds reported in these studies were targeted to the reported hydrophobic hotspots 1 and 2. In a subsequent publication, the same group also reported that BMS compounds, specifically BMS-200, based on 2,3-dihydro-1,4-benzdioxinyl group induced an enlarged interaction interface that results in the open “face-back” tunnel through the PD-L1 dimer (38). This result indicates that BMS-200 induces conformational changes in PD-L1, and the ligand binding site of PD-L1 to be more flexible than it was previously seen. The crystal structure of the less cytotoxic compounds (BMS-1001 and 1166) from the BMS compounds series indicated that the deep hydrophobic pocket harboring BMS-202 is transformed into a tunnel in the PD-L1/BMS-1166 structure by rotation of the Tyr56 sidechain, which may be responsible for increased potency of the compounds compared to their predecessors (98). Using multiple molecular modeling methods, computational insights into the small molecule induced PD-L1 dimerization, suggesting the tendency of BMS small-molecule inhibitors to interact with one PD-L1 monomer first followed by dimer formation for an advantage of stability has been provided (105).

**Figure 6:**
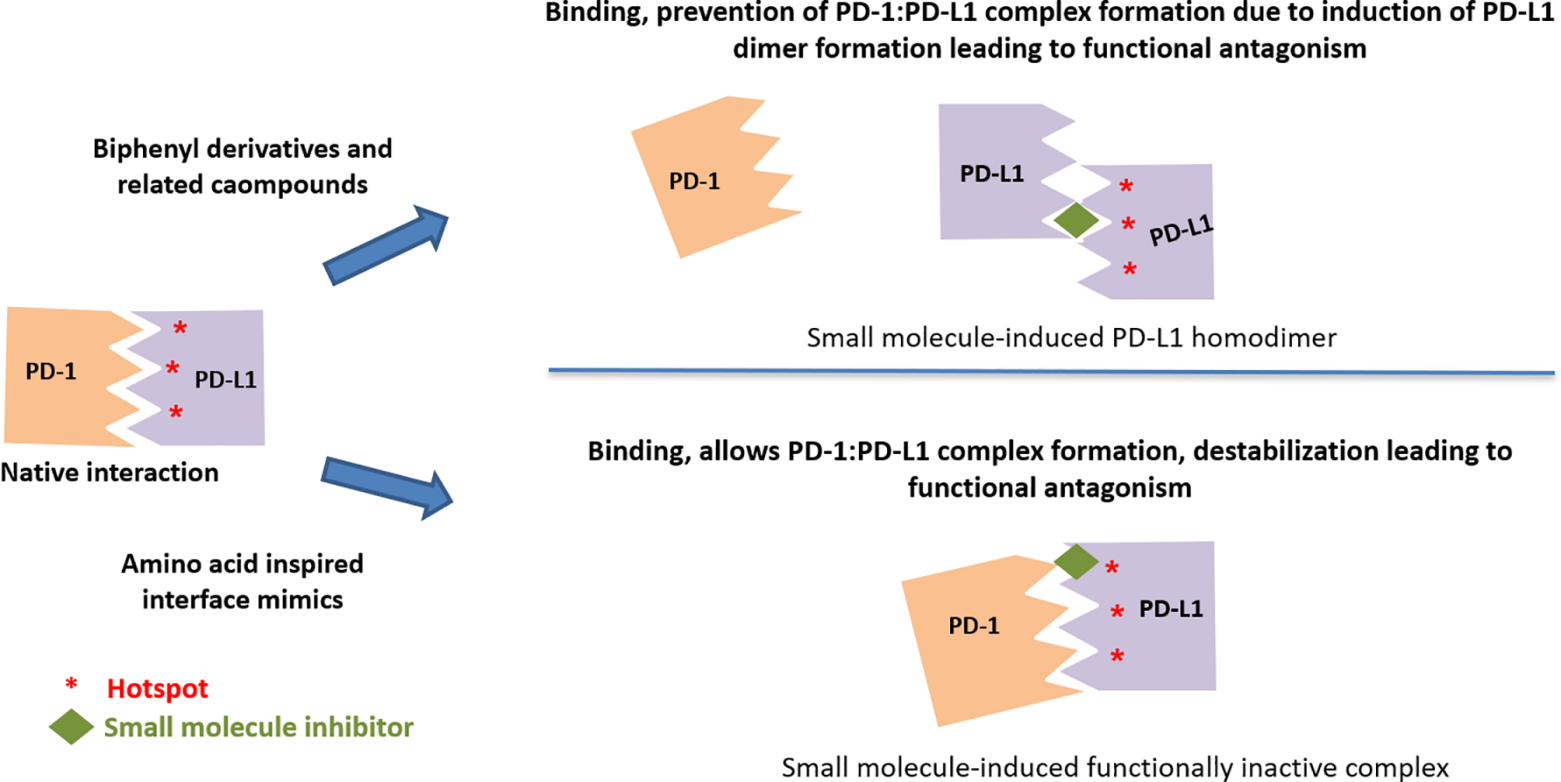
Two distinct mechanisms leading to functional antagonism with reported small molecule PD-L1 inhibitors.

The more active (in both binding and functional assays) C_2_-symmetic inhibitor Compound LH1307 was found to extend across the AFGCC’ faces of the PD-L1 proteins, forming an extended hydrophobic tunnel through the PD-L1 homodimer (42). In this molecule, the biphenyl moiety helped in anchoring the compound in the hydrophobic core of the homodimer, while the polar groups in the solvent exposed helped in bringing the two PD-L1 monomers through a network of hydrogen bonds and electrostatic interactions with the polar residues on the AB and CC’ strands of the PD-L1. Greater potency of such symmetrical compound was due to the binding two equivalent sites in the PD-L1 homodimer by virtue of its C_2_-symmetric nature (42). Incyte and Arbutus have also utilized the inherent C2-symmetry in the PD-L1 dimer by symmetrizing their molecules to achieve greater potency (**Figure 6**).

A recent review (106) summarizes the phenomenon of drug-induced protein dimerization, as the mechanism to modulate the target activity is not unique to PD-L1. Other examples of drug-induced protein homodimer stabilization include compounds that stabilize Max and TLR2 homodimers. In these examples, the same dimer sequestration principle occurs with the compounds, which shape an interfacial binding tunnel between two monomeric protein units. Interestingly, compounds used to promote homo-dimerization of matrix metalloproteinases for crystallographic studies also include a biphenyl unit (107).

### Blocking of PD-L1 Export From Endoplasmic Reticulum to Golgi

A cellular mechanism for the functional antagonism of the biphenyl compound BMS-1166 based on the role of glycosylation on the critical residues of PD-L1 protein has also been proposed (108). The four N-glycosylation sites (Asn35, Asn192, Asn 200, and Asn 219) present in the extracellular domain of PD-L1 is necessary for the stability of ligand protein, and except Asn 35, all other glycosylation sites are critical for its interaction with receptor (90, 109). BMS-1166 partially and specifically inhibits PD-L1 glycosylation and functionally inactivates PD-L1 by blocking its export from the endoplasmic reticulum to Golgi (108). It is not known if the newer C_2_ symmetric compounds also have an impact on PD-L1 by this mechanism.

### Induction of PD-L1 Dimerization and Internalization

Induction of a two-step internalization process as the mechanism of action for a C_2_-skeleton biphenyl compounds has recently been published by the Arbutus group (44). In this process, Compound ARB-272572 inhibits the PD-1/PD-L1 axis by inducing cell surface PD-L1 dimerization *via* cis-interacting homodimers triggering a rapid loss of cell surface PD-L1 by rapid internalization into the cytosol, thereby preventing any further interaction with PD-1-expressing cell types. The formation of homodimer as illustrated by a crystal structure where ARB 272542 (44) bound within a hydrophobic pocket created between two PD-L1 proteins followed by rapid internalization into the cytosol is reported to be the primary mechanism contributing to cellular potency. Similar to the publication by Arbutus, Incyte had previously described inhibitor-induced internalization, but the structure of the specific compound was not revealed (110). Apart from the clinical candidate, Incyte also reported INCB-090244, which binds and internalizes surface PD-L1 *in vivo* in a dose-dependent manner, and orally dosed INCB-090244 exhibits single agent activity and increases infiltrating T cells in two distinct humanized mouse models (110). Although the induction of PD-L1 internalization has been reported only with C_2_-symmetric molecules., it is not clear if the C_2_ symmetry and the resulting interaction are prerequisites for inducing internalization.

In the mechanism of action of PD-L1 inhibitor that induces internalization, PD-L1 is no longer available on the cell surface not only to interact with its cognate receptor PD-1 but also for its other reported binding partner Cluster of Differentiation 24 (CD80) (111). This may have unwanted consequences based on the reported finding indicating that PD-L1 also exerts an immunostimulatory effect by repressing the CTLA-4 pathway (112). The reduced cell surface PD-L1 levels could lead to greater availability of CD80 to its cognate receptor CTLA-4 leading to immune suppression. Hence, the disappearance of PD-L1 on the cell surface because of the internalization and its functional consequence may need to be studied further, as the checkpoint biology is still emerging (113).

### Binding to PD-L1 Without Interference in the PD1:PD-L1 Complex Formation

Lack of binding of amino-acids-inspired interface mimic CA-170 to either PD-1 or PD-L1 or disruption of PD-1:PD-L1 complex in various biophysical and biochemical assays has been discussed in the literature along with postulating alternative unknown mechanism of action for these compounds (82, 83, 93). In view of the use of proteins expressed in heterologous expression systems lacking appropriate post-translational modifications, recently, the binding of CA-170 to PD-L1 has been established by cellular NMR spectroscopy using full-length PD-L1 expressed in mammalian cells (19). Compared to the biphenyl-based small molecule inhibitors (38, 98), CA-170 is highly polar and thus likely interacts with PD-L1 at a solvent exposed region. Observed functional antagonism in PD-L1 signaling and direct binding to PD-L1 in the cellular context without the disruption of the PD1:PD-L1 complex support the formation of a defective ternary complex as the mechanism of action of CA-170 (19) (**Figure 6**). This mode of action of CA-170 is analogous to that of two reported anti-PD1 antibodies that antagonize PD-1 signaling without interfering in PD-1:PD-L1 complex formation (114, 115).

## Clinical Development of PD-1 Small Molecule Inhibitors

CA-170 was the first orally available small molecule PD-L1 inhibitor to enter the clinical trials in 2016, and it is currently in Phase 2b/3 trials (co-development by Curis and Aurigene). The clinical trials of Incyte compound INCB-086550 was initiated in 2018, and Phase 1 is expected to be completed by 2022. The details of all small molecule agents in the clinic with most recent findings are consolidated in **Table 3**.

**Table 3:** Details of small molecules in clinical trials.

Sl. no.	Drug/company	Dosing	Indications	Current status	Details of recent status
1	**CA-170**	50 mg QD to 1,200 mg	Lymphoma; advanced solid tumors	Phase 2b/3	In Phase 2 studies, ORR of 30% in classical Hodgkin lymphoma (based on Lugano criteria) and a CBR of >85% at a daily dose of 400 mg and PFS of 19.6 weeks in advanced (stage 4) non-squamous NSCLC (101) CTRI/2017/12/011026
	Aurigene/Curis				Phase2b/3 studies: Phase 2b/3 randomized study of CA-170 in patients with non-squamous non-small cell lung cancer CTRI/2020/07/026870
2	**INCB-086550**	QD (100, 200 mg) or BID (200, 400 mg).	NSCLC urothelial cancer	Phase 2	Phase 1 Study Exploring the Safety, Tolerability, PK and PD (NCT03762447).
	Incyte Corp		Renal cell carcinoma		A dose-related 1.2-fold increase in the plasma concentration of soluble target (PD-L1); 3.4-fold increase in IFN- γ; 1.3- and 1.4-fold of CXCL9 and CXCL10, observed post-treatment (116, 086550).
			Hepatocellular carcinoma		Phase 2: Open-label, non-randomized study to evaluate the efficacy and safety (NCT04629339); INCB-086550 will be administered orally twice a day.
			Melanoma		
3	**MX-10181**	Dose not disclosed	Advanced solid tumor; cancer	Phase 1	In February 2021, an implied trial approval was granted for advanced solid tumor.
	Maxinovel Pharma				Part 1: Dose escalation, MAX-10181 once or twice daily with dose modifications based on tolerability criteria.
					Part 2: Dose expansion, Recommended doses from Part 1 (NCT04122339).
4	**GS-4224**	Starting at 400 mg once a day (QD). Subsequent doses of 700 mg QD, 1,000 mg QD, 1,500 mg QD, and 1,000 mg twice a day (BID)	Advanced solid tumor; Hepatitis B virus infection	Phase 1	In March 2021, Gilead Sciences terminated a phase Ib/II trial to evaluate the safety, tolerability, pharmacokinetics, and efficacy of GS 4224 in patients with advanced solid tumors (NCT04049617).
	Gilead Sciences				
5	**IMMH-010**	Starting at 60 mg QD. Subsequent doses of 120, 240, and 360 mg QD	Malignant neoplasms	Phase 1	Trial approved in April 2020 and the study is not yet recruited.
	Tianjin Chasesun Pharmaceutical Co., LTD				To determine MTD and RP2D and to evaluate the effects of food on the pharmacokinetic profiles after single dose of IMMH-010 in patients with advanced solid tumors (NCT04343859).

### Considerations in Small Molecule Clinical Development

Several unique features of small molecule agents targeting PD-L1 compared to antibodies against PD-1 and PD-L1 emphasize the potential need to adapt strategies that are different than those that have worked well for approved PD-1 and PD-L1 antibodies. Target engagement analysis in circulation that is widely used for antibody-based therapeutics may not work for small molecule reversible agents because of their distinct binding kinetics including faster off-rate. In the absence of the direct target engagement, dose selection for expansion studies my need to be decided based on the exposure and its correlation with pharmacodynamic effects observed in both preclinical and clinical setting.

Because of the substantially smaller size of small molecule PD-L1 agents, a significantly higher number of molecules (300´ or more) are delivered compared an antibody-based agents at the same dosage level. If the potency of a small molecule PD-L1 agent is similar to anti-PD-1/PD-L1 antibody in primary T-cell-based functional assays, specifically in the rescue of inhibition by PD-L1 (as has been shown for amino-acids-inspired interface mimic CA-170 and several biphenyl derivatives), it is important to think through the consequence of achieving very high molar exposure in the clinic relative to antibodies. As discussed earlier in this article, because of the observed bell-shaped T-cell activation response in preclinical studies, the conventional approach in oncology such as first determining the maximum tolerated dose (MTD), followed by dose expansion at the MTD, may not be ideal and in fact may be highly counterproductive for an immune-activating agent.

Classically, dose–response to drugs is generally considered to be sigmoidal in nature where the biological effect increases linearly with dose until the threshold concentrations whereby inhibition proceeds in a linear fashion until saturation is achieved. However, there are examples in various classes of drugs where the dose–response curve deviates from the sigmoidal curve and exhibits a hormetic (bell-, U-, or J-shaped) dose–response. A classic example of bell-shaped clinical response for an immunomodulating agent was reported for IFN-γ several decades ago with efforts that resulted in identifying optimal immunomodulatory dose (OID) (117). Preclinical and clinical studies have suggested that the OID of may not be the clinical MTD (118–120). Hence, the clinical trials carried out with IFN-γ at MTD resulting in low response rate was attributed to failure in treating the patients at an optimal OID. Such bell-shared clinical response has also been noted with small molecule immunotherapeutic agents such as STING agonists (121) or TLR agonists (122). It is also worth noting that a bell-shaped clinical response has also been reported for anti-PD1 antibody nivolumab (123, 124), a finding that is not widely highlighted.

Clinical exposure-efficacy data of small molecule PD-L1 agents are slowly unfolding, and they indicate an inverse dose relationship in efficacy. Radhakrishnan et al. reported the clinical benefit for CA-170 at a dosage of 400 mg QD compared to 800 mg QD dose (101). Analysis of safety profile of the drug also indicated higher incidence of irAEs at the lower dosage of 400 mg. Additionally, there was a strong overall trend of improved CBR and PFS with 400 mg when compared to the higher dose (800 mg) in both lung cancer and Hodgkin lymphoma. These agree with preclinical findings showing a bell-shaped curve of immune activation likely due to activation-induced cell death with CA-170 (discussed earlier in this article). Observed clinical activity of CA-170 at lower dose is also consistent with the exposure achieved at optimal dose of 10 mg/kg in preclinical models (19). A recent preclinical study also reported potent anticancer efficacy of CA-170 at a dose of 10 mg/kg in carcinogen-induced mouse lung tumorigenesis model (20). Clinical efficacy data are not yet reported for INCB-086550, but peripheral pharmacodynamic activity in Phase 1 studies is reported for a low dose (100, 200 mg QD dosing or 200, 400 mg BID dosing), and Phase 2 dosing is currently ongoing (dosage not disclosed) orally twice a day.

The clinical and preclinical data of CA-170 discussed here highlight the need to adjust the optimal immunomodulatory dose for attaining clinical efficacy. In the absence of any tolerability issues, the recommended Phase 2 dose (RP2D) for a small molecule PD-L1 agent is likely the dose that achieves a reliable immune response/pharmacodynamics (assessed by monitoring T cells or other immune cells in the tumor microenvironment) and hints of clinical efficacy or exposure that shows anti-tumor efficacy in preclinical models. Further studies on the dose−efficacy relationship and the underlying mechanisms need to be understood further to achieve complete benefit of these agents in clinic.

Since most of the reported small molecule inhibitors target PD-L1 as opposed to PD-1, with the exception of limited reports (125), clinical indications in which anti-PD-L1 antibodies have shown good efficacy could be more appropriate for PD-L1 small molecule agents. Unlike PD-1 antibodies that block signaling from both PD-L1 and PD-L2, PD-L1 antibodies do not impact PD-L2 signaling. On the other hand, PD-L1 antibodies also block the interaction of PD-L1 with CD80. Even though there is a significant overlap in the approved clinical indications for PD-1 antibodies versus PD-L1 antibodies, there are specific indications including Hodgkin lymphoma, head and neck cancer, and colorectal cancers in which only PD-1 antibodies are approved. In the absence of head-to-head comparison and the dependence on cross-study comparisons, it is not clear if these differences are due to differential biology expected from PD-1-targeted agents versus those targeting PD-L1. However, these points may need to be taken into consideration during the development of PD-L1-targeted agents.

## Conclusions and Future Perspectives

With the realization that small molecule agents targeting PD-1:PD-L1 immune checkpoint pathway offer distinct advantages, there has been a burst of publications describing several agents targeting PD-L1. Interestingly, while most of these agents interact with PD-L1 and result in the desired phenotype, there has been limited report(s) of small molecule agents binding to PD1 or both PD1 and PD-L1. Because of several elegant studies, the mechanisms of action of PD-L1 agents are unravelling, but we still do not know if targeting PD-1 would be more advantageous. While most of the reported agents derived from the biphenyl scaffold perform very well in the binding and PD-1:PD-L1 disruption assays by inducing dimerization of PD-L1, not all of them show potent functionality in cellular systems. Among the functional assays evaluated, PBMC or primary T-cell-based assays, specifically those in which rescue of inhibition by PD-L1 is monitored, show a better correlation with potency in biochemical assays for disruption of PD-1:PD-L1 interaction. Disconnection in several other functional assays is likely contributed by differences in which these molecules encounter the target on immune cells in a physiological context, which emphasizes the need to use appropriate functional assays in the discovery. Functional assays using primary T cells from human and preclinical species provide their cross-species activity and valuable information for clinical translation with respect to exposure that would likely lead to optimal immune activation. While there is substantial progress in identifying PD-L1 targeted small molecule agents, it is intriguing to note that not many have attempted to characterize the interaction of the reported PD-L1 agents with PD-L2. Similarly, the consequences of the binding to PD-L1 on PD-L1’s interaction with CD80 are yet to be characterized for most of the reported PD-L1 agents. Ultimately, the utility of the PD-L1-targeted small molecule agents needs to be delineated by clinical studies, and in this regard, it is encouraging to note that clinical studies are progressing with a few of the molecules. Early clinical data suggest a need for a precise determination of the dose providing optimal immune activation for dose expansion without escalating it to MTD as typically followed for most cytotoxic or targeted oncology drugs. Clinical success with small molecule PD-L1 inhibitors either as a single agent or in combination with current standard of care is eagerly awaited towards fully harnessing this important therapeutic modality.

## Author Contributions

PS and MR performed the literature review and wrote the manuscript. All authors contributed to the article and approved the submitted version.

## Conflict of Interest

Authors are present or previous employees of Aurigene Discovery Technologies Limited. PS and MR are inventors on several patent applications related to immune checkpoint inhibitors described in this manuscript. MR is the CEO of Aurigene Discovery Technologies Limited, a company that is co-developing CA-170 for cancer therapy.

## Publisher’s Note

All claims expressed in this article are solely those of the authors and do not necessarily represent those of their affiliated organizations, or those of the publisher, the editors and the reviewers. Any product that may be evaluated in this article, or claim that may be made by its manufacturer, is not guaranteed or endorsed by the publisher.

## References

[1] Ledford H Else H Warren M. Cancer Immunologists Scoop Medicine Nobel Prize. Nature (2018) 562(7725):20–1. doi: 10.1038/d41586-018-06751-0 30279600

[2] Vaddepally RK Kharel P Pandey R Garje R Chandra AB. Review of Indications of FDA-Approved Immune Checkpoint Inhibitors Per NCCN Guidelines With the Level of Evidence. Cancers (2020) 12(3):735. doi: 10.3390/cancers12030738 PMC7140028 32245016

[3] Pardoll DM. The Blockade of Immune Checkpoints in Cancer Immunotherapy. Nat Rev Cancer (2012) 12(4):252–64. doi: 10.1038/nrc3239 PMC4856023 22437870

[4] Ghosh A Barba P Perales M-A. Checkpoint Inhibitors in AML: Are We There Yet? Br J Haematol (2020) 188(1):159–675. doi: 10.1111/bjh.16358 31808941 PMC9549702

[5] Naidoo J Page DB Li BT Connell LC Schindler K Lacouture ME. Toxicities of the Anti-PD-1 and Anti-PD-L1 Immune Checkpoint Antibodies. Ann Oncol (2015) 26(12):2375–91. doi: 10.1093/annonc/mdv383 PMC6267867 26371282

[6] Larkin J Chiarion-Sileni V Gonzalez R Grob JJ Cowey CL Lao CD. Combined Nivolumab and Ipilimumab or Monotherapy in Untreated Melanoma. New Engl J Med (2015) 373(1):23–34. doi: 10.1056/NEJMoa1504030 26027431 PMC5698905

[7] Wolchok JD Chiarion-Sileni V Gonzalez R Rutkowski P Grob J-J Cowey CL. Overall Survival With Combined Nivolumab and Ipilimumab in Advanced Melanoma. New Engl J Med (2017) 377(14):1345–56. doi: 10.1056/NEJMoa1709684 PMC5706778 28889792

[8] Redelman-Sidi G Michielin O Cervera C Ribi C Aguado JM Fernández-Ruiz M. ESCMID Study Group for Infections in Compromised Hosts (ESGICH) Consensus Document on the Safety of Targeted and Biological Therapies: An Infectious Diseases Perspective (Immune Checkpoint Inhibitors, Cell Adhesion Inhibitors, Sphingosine-1-Phosphate Receptor Modulators and Proteasome Inhibitors). Clin Microbiol Infect (2018) 24:S95–107. doi: 10.1016/j.cmi.2018.01.030 29427804 PMC5971148

[9] Brahmer JR Drake CG Wollner I Powderly JD Picus J Sharfman WH. Phase I Study of Single-Agent Anti-Programmed Death-1 (MDX-1106) in Refractory Solid Tumors: Safety, Clinical Activity, Pharmacodynamics, and Immunologic Correlates. J Clin Oncol (2010) 28(19):3167–75. doi: 10.1200/JCO.2009.26.7609 PMC4834717 20516446

[10] Brahmer JR Tykodi SS Chow LQM Hwu W-J Topalian SL Hwu P. Safety and Activity of Anti-PD-L1 Antibody in Patients With Advanced Cancer. New Engl J Med (2012) 366(26):2455–65. doi: 10.1056/NEJMoa1200694 PMC3563263 22658128

[11] Topalian SL Hodi FS Brahmer JR Gettinger SN Smith DC McDermott DF. Safety, Activity, and Immune Correlates of Anti-PD-1 Antibody in Cancer. New Engl J Med (2012) 366(26):2443–54. doi: 10.1056/NEJMoa1200690 PMC3544539 22658127

[12] Koyama S Akbay EA Li YY Herter-Sprie GS Buczkowski KA Richards WG. Adaptive Resistance to Therapeutic PD-1 Blockade Is Associated With Upregulation of Alternative Immune Checkpoints. Nat Commun (2016) 7:10501. doi: 10.1038/ncomms10501 26883990 PMC4757784

[13] Gao J Ward JF Pettaway CA Shi LZ Subudhi SK Vence LM. VISTA Is an Inhibitory Immune Checkpoint That Is Increased After Ipilimumab Therapy in Patients With Prostate Cancer. Nat Med (2017) 23(5):551–55. doi: 10.1038/nm.4308 PMC5466900 28346412

[14] Jain RK. Physiological Barriers to Delivery of Monoclonal Antibodies and Other Macromolecules in Tumors. Cancer Res (1990) 50(3 Suppl):814s–19. 2404582

[15] Andrews A. Treating With Checkpoint Inhibitors-Figure $1 Million Per Patient. Am Health Drug Benefits (2015) 8(Spec Issue):9–9. 26380599 PMC4570079

[16] Imai K Takaoka A. Comparing Antibody and Small-Molecule Therapies for Cancer. Nat Rev Cancer (2006) 6(9):714–275. doi: 10.1038/nrc1913 16929325

[17] Yabroff KR Warren JL Knopf K Davis WW Brown ML. Estimating Patient Time Costs Associated With Colorectal Cancer Care. Med Care (2005) 43(7):640–485. doi: 10.1097/01.mlr.0000167177.45020.4a 15970778

[18] Chames P Regenmortel MV Weiss E Baty D. Therapeutic Antibodies: Successes, Limitations and Hopes for the Future: Therapeutic Antibodies: An Update. Br J Pharmacol (2009) 157(2):220–335. doi: 10.1111/j.1476-5381.2009.00190.x 19459844 PMC2697811

[19] Sasikumar PG Sudarshan NS Adurthi S Ramachandra RK Samiulla DS Lakshminarasimhan A. PD-1 Derived CA-170 Is an Oral Immune Checkpoint Inhibitor That Exhibits Preclinical Anti-Tumor Efficacy. Commun Biol (2021) 4(1):699. doi: 10.1038/s42003-021-02191-1 34103659 PMC8187357

[20] Pan J Chen Y Zhang Q Khatun A Palen K Xin G. Inhibition of Lung Tumorigenesis by a Small Molecule CA170 Targeting the Immune Checkpoint Protein VISTA. Commun Biol (2021) 4(1):906. doi: 10.1038/s42003-021-02381-x 34302042 PMC8302676

[21] Findlay M von Minckwitz G Wardley A. Effective Oral Chemotherapy for Breast Cancer: Pillars of Strength. Ann Oncol (2008) 19(2):212–22. doi: 10.1093/annonc/mdm285 18006898

[22] Makurvet FD. Biologics vs. Small Molecules: Drug Costs and Patient Access. Med Drug Discovery (2021) 9:100075. doi: 10.1016/j.medidd.2020.100075

[23] Schneider C. Monoclonal Antibodies - Regulatory Challenges. Curr Pharm Biotechnol (2008) 9(6):431–38. doi: 10.2174/138920108786786394 19075683

[24] Vandivort TC Horton DB Johnson SB. Regulatory and Strategic Considerations for Addressing Immunogenicity and Related Responses in Biopharmaceutical Development Programs. J Clin Trans Sci (2020) 4(6):547–55. doi: 10.1017/cts.2020.493 PMC8057416 33948231

[25] Smith MC Gestwicki JE. Features of Protein–Protein Interactions That Translate Into Potent Inhibitors: Topology, Surface Area and Affinity. Expert Rev Mol Med (2012) 14:e16. doi: 10.1017/erm.2012.10 22831787 PMC3591511

[26] Ivanov AA Khuri FR Fu H. Targeting Protein–Protein Interactions as an Anticancer Strategy. Trends Pharmacol Sci (2013) 34(7):393–4005. doi: 10.1016/j.tips.2013.04.007 23725674 PMC3773978

[27] Guo W Wisniewski JA Ji H. Hot Spot-Based Design of Small-Molecule Inhibitors for Protein–Protein Interactions. Bioorg Med Chem Lett (2014) 24(11):2546–545. doi: 10.1016/j.bmcl.2014.03.095 24751445

[28] Schmidtke P Barril X. Understanding and Predicting Druggability. A High-Throughput Method for Detection of Drug Binding Sites. J Med Chem (2010) 53(15):5858–675. doi: 10.1021/jm100574m 20684613

[29] Reichmann D Rahat O Albeck S Meged R Dym O Schreiber G. The Modular Architecture of Protein-Protein Binding Interfaces. Proc Natl Acad Sci (2005) 102(1):57–62. doi: 10.1073/pnas.0407280102 15618400 PMC544062

[30] Moza B Buonpane RA Zhu P Herfst CA Rahman AKMN-u McCormick JK. Long-Range Cooperative Binding Effects in a T Cell Receptor Variable Domain. Proc Natl Acad Sci (2006) 103(26):9867–725. doi: 10.1073/pnas.0600220103 PMC1502545 16788072

[31] Ofran Y Rost B. Protein–Protein Interaction Hotspots Carved Into Sequences. In: Valencia A, editor. PLoS Computational Biology, vol. 3 (2007). doi: 10.1371/journal.pcbi.0030119 PMC1914369 17630824

[32] Keskin O Ma B Nussinov R. Hot Regions in Protein–Protein Interactions: The Organization and Contribution of Structurally Conserved Hot Spot Residues. J Mol Biol (2005) 345(5):1281–945. doi: 10.1016/j.jmb.2004.10.077 15644221

[33] Bogan AA Thorn KS. Anatomy of Hot Spots in Protein Interfaces. J Mol Biol (1998) 280(1):1–95. doi: 10.1006/jmbi.1998.1843 9653027

[34] Darnell SJ LeGault L Mitchell JC. KFC Server: Interactive Forecasting of Protein Interaction Hot Spots. Nucleic Acids Res (2008) 36:W265–69. doi: 10.1093/nar/gkn346 PMC2447760 18539611

[35] Zak KM Kitel R Przetocka S Golik P Guzik K Musielak B. Structure of the Complex of Human Programmed Death 1, PD-1, and Its Ligand PD-L1. Struct (London England: 1993) (2015) 23(12):2341–485. doi: 10.1016/j.str.2015.09.010 PMC4752817 26602187

[36] Huang D Wen W Liu X Li Y Zhang JZH. Computational Analysis of Hot Spots and Binding Mechanism in the PD-1/PD-L1 Interaction. RSC Adv (2019) 9(26):14944–565. doi: 10.1039/C9RA01369E PMC9064197 35516311

[37] Lipinski CA Lombardo F Dominy BW Feeney PJ. Experimental and Computational Approaches to Estimate Solubility and Permeability in Drug Discovery and Development Settings 1PII of Original Article: S0169-409x(96)00423-1. Article Originally Published Adv Drug Deliv Rev (2001) 23(1997):3–25. doi: 10.1016/S0169-409X(00)00129-0 11259830

[38] Guzik K Zak KM Grudnik P Magiera K Musielak B Törner R. Small-Molecule Inhibitors of the Programmed Cell Death-1/Programmed Death-Ligand 1 (PD-1/PD-L1) Interaction *via* Transiently Induced Protein States and Dimerization of PD-L1. J Med Chem (2017) 60(13):5857–675. doi: 10.1021/acs.jmedchem.7b00293 28613862

[39] Guzik K Tomala M Muszak D Konieczny M Hec A Błaszkiewicz U. Development of the Inhibitors That Target the PD-1/PD-L1 Interaction—A Brief Look at Progress on Small Molecules, Peptides and Macrocycles. Molecules (2019) 24(11):20715. doi: 10.3390/molecules24112071 PMC6600339 31151293

[40] Sasikumar PG Ramachandra M. Small-Molecule Immune Checkpoint Inhibitors Targeting PD-1/PD-L1 and Other Emerging Checkpoint Pathways. BioDrugs (2018) 32(5):481–975. doi: 10.1007/s40259-018-0303-4 30168070

[41] Wu X Meng Y Liu L Gong G Zhang H Hou Y. Insights Into Non-Peptide Small-Molecule Inhibitors of the PD-1/PD-L1 Interaction: Development and Perspective. Bioorg Med Chem (2021) 33(March):116038. doi: 10.1016/j.bmc.2021.116038 33517226

[42] Basu S Yang J Xu B Magiera-Mularz K Skalniak L Musielak B. Design, Synthesis, Evaluation, and Structural Studies of C2-Symmetric Small Molecule Inhibitors of Programmed Cell Death-1/Programmed Death-Ligand 1 Protein–Protein Interaction. J Med Chem (2019) 62(15):7250–635. doi: 10.1021/acs.jmedchem.9b00795 31298541

[43] Kawashita S Aoyagi K Yamanaka H Hantani R Naruoka S Tanimoto A. Symmetry-Based Ligand Design and Evaluation of Small Molecule Inhibitors of Programmed Cell Death-1/Programmed Death-Ligand 1 Interaction. Bioorg Med Chem Lett (2019) 29(17):2464–67. doi: 10.1016/j.bmcl.2019.07.027 31351692

[44] Park J-J Thi EP Carpio VH Bi Y Cole AG Dorsey BD. Checkpoint Inhibition Through Small Molecule-Induced Internalization of Programmed Death-Ligand 1. Nat Commun (2021) 12(1):1222. doi: 10.1038/s41467-021-21410-1 33619272 PMC7900207

[45] Feng Z Chen X Yang Y Zheng Y Lai F Ji M. Nicotinyl Alcohol Ether Derivative, Preparation Method Therefor, and Pharmaceutical Composition and Uses Thereof. United States: Patent and Trademark Office (USPTO)(2021). Available at: https://patents.google.com/patent/US10975049B2/en. US10975049B2.

[46] Tianjin Chasesun Pharmaceutical Co., LTD. A Phase I Clinical Trial of IMMH-010 in Patients With Advanced Malignant Solid Tumors. In: Clinical trial registration NCT04343859. clinicaltrials.gov (2020). Available at: https://clinicaltrials.gov/ct2/show/NCT04343859.

[47] Jiang J Zou X Liu Y Liu X Dong K Yao X. Simultaneous Determination of a Novel PD-L1 Inhibitor, IMMH-010, and Its Active Metabolite, YPD-29B, in Rat Biological Matrices by Polarity-Switching Liquid Chromatography-Tandem Mass Spectrometry: Application to ADME Studies. Front Pharmacol (2021) 12:677120. doi: 10.3389/fphar.2021.677120 34234673 PMC8256334

[48] Cheng B Ren Y Cao H Chen J. Discovery of Novel Resorcinol Diphenyl Ether-Based PROTAC-Like Molecules as Dual Inhibitors and Degraders of PD-L1. Eur J Med Chem (2020) 199:112377. doi: 10.1016/j.ejmech.2020.112377 32388281

[49] Yang Y Wang K Chen H Feng Z. Design, Synthesis, Evaluation, and SAR of 4-Phenylindoline Derivatives, a Novel Class of Small-Molecule Inhibitors of the Programmed Cell Death-1/Programmed Cell Death-Ligand 1 (PD-1/PD-L1) Interaction. Eur J Med Chem (2021) 211(February):113001. doi: 10.1016/j.ejmech.2020.113001 33272783

[50] Chen A Wu D-L Shi J Narva S Zhao X-Y Wu Y-L. Design, Synthesis and Biological Evaluation of 2-Methyl-(1,1′-Biphenyl)-Pyrimidine Conjugates. Bioorg Med Chem Lett (2020) 30(16):1273285. doi: 10.1016/j.bmcl.2020.127328 32631533

[51] Wang F Ye W Wang S He Y Zhong H Wang Y. Discovery of a New Inhibitor Targeting PD-L1 for Cancer Immunotherapy. Neoplasia (2021) 23(3):281–93. doi: 10.1016/j.neo.2021.01.001 PMC7851350 33529880

[52] Liu C Zhou F Yan Z Shen L Zhang X He F. Discovery of a Novel, Potent and Selective Small-Molecule Inhibitor of PD-1/PD-L1 Interaction With Robust *in Vivo* Anti-Tumour Efficacy. Br J Pharmacol (2021) 1778(13):2651. doi: 10.1111/bph.15457 33768523

[53] Dai X Wang K Chen H Huang X Feng Z. Design, Synthesis, and Biological Evaluation of 1-Methyl-1h-Pyrazolo[4,3-B]Pyridine Derivatives as Novel Small-Molecule Inhibitors Targeting the PD-1/PD-L1 Interaction. Bioorg Chem (2021) 114:105034. doi: 10.1016/j.bioorg.2021.105034 34116264

[54] Guo J Luo L Wang Z Hu N Wang W Xie F. Design, Synthesis, and Biological Evaluation of Linear Aliphatic Amine-Linked Triaryl Derivatives as Potent Small-Molecule Inhibitors of the Programmed Cell Death-1/Programmed Cell Death-Ligand 1 Interaction With Promising Antitumor Effects *In Vivo* . J Med Chem (2020) 63(22):13825–505. doi: 10.1021/acs.jmedchem.0c01329 33186040

[55] Wang T Cai S Wang M Zhang W Zhang K Chen D. Novel Biphenyl Pyridines as Potent Small-Molecule Inhibitors Targeting the Programmed Cell Death-1/Programmed Cell Death-Ligand 1 Interaction. J Med Chem (2021) 64(11):7390–403. doi: 10.1021/acs.jmedchem.1c00010 34056906

[56] OuYang Y Gao J Zhao L Lu J Zhong H Tang H. Design, Synthesis, and Evaluation of *O* -(Biphenyl-3-Ylmethoxy)Nitrophenyl Derivatives as PD-1/PD-L1 Inhibitors With Potent Anticancer Efficacy *In Vivo* . J Med Chem (2021) 64(11):7646–66. doi: 10.1021/acs.jmedchem.1c00370 34037385

[57] Muszak D Surmiak E Plewka J Magiera-Mularz K Kocik-Krol J Musielak B. Terphenyl-Based Small-Molecule Inhibitors of Programmed Cell Death-1/Programmed Death-Ligand 1 Protein–Protein Interaction. J Med Chem (2021) 64(15):11614–36. doi: 10.1021/acs.jmedchem.1c00957 PMC8365601 34313116

[58] Collins M Ling V Carreno BM. The B7 Family of Immune-Regulatory Ligands. Genome Biol (2005) 6(6):2235. doi: 10.1186/gb-2005-6-6-223 PMC1175965 15960813

[59] Sasikumar PG Ramachandra M. Peptide and Peptide-Inspired Checkpoint Inhibitors: Protein Fragments to Cancer Immunotherapy. Med Drug Discovery (2020) 8:100073. doi: 10.1016/j.medidd.2020.100073

[60] Giannis A Kolter T. Peptidomimetics for Receptor Ligands?Discovery, Development, and Medical Perspectives. Angew Chem Int Ed English (1993) 32(9):1244–675. doi: 10.1002/anie.199312441

[61] Pelay-Gimeno M Glas A Koch O Grossmann TN. Structure-Based Design of Inhibitors of Protein-Protein Interactions: Mimicking Peptide Binding Epitopes. Angew Chem Int Ed (2015) 54(31):8896–89275. doi: 10.1002/anie.201412070 PMC4557054 26119925

[62] Ripka AS Rich DH. Peptidomimetic Design. Curr Opin Chem Biol (1998) 2(4):441–525. doi: 10.1016/S1367-5931(98)80119-1 9736916

[63] Azzarito V Long K Murphy NS Wilson AJ. Inhibition of α-Helix-Mediated Protein–Protein Interactions Using Designed Molecules. Nat Chem (2013) 5(3):161–735. doi: 10.1038/nchem.1568 23422557

[64] Akram ON DeGraff DJ Sheehan JH Tilley WD Matusik RJ Ahn J-M. Tailoring Peptidomimetics for Targeting Protein–Protein Interactions. Mol Cancer Res (2014) 12(7):967–785. doi: 10.1158/1541-7786.MCR-13-0611 24642350

[65] Sasikumar PGN Ramachandra M Naremaddepalli SS. Peptidomimetic Compounds as Immunomodulators. World Intellectual Property Organization (2013). Available at: https://patents.google.com/patent/WO2013132317A1/en.

[66] Sasikumar PGN Ramachandra M Appukuttan P Naremaddepalli SSS. 3-Substituted-1,2,4-Oxadiazole and Thiadiazole Compounds as Immunomodulators. (2016). Available at: https://patents.google.com/patent/WO2016142886A3/en

[67] Sasikumar PGN Ramachandra M Naremaddepalli SSS. 1,2,4-Oxadiazole Derivatives as Immunomodulators. World Intellectual Property Organization (2015). Available at: https://patents.google.com/patent/WO2015033299A1/en?oq=WO2015033299.

[68] Sasikumar PGN Ramachandra M Naremaddepalli SSS. 1,3,4-Oxadiazole and 1,3,4-Thiadiazole Derivatives as Immunomodulators. World Intellectual Property Organization WO2015033301A1. International Bureau. (2015). Available at: https://patents.google.com/patent/WO2015033301A1/en?oq=WO2015033301.

[69] Xu Y Lu H Hai H Lindang. Cyclic Compounds Inhibiting Programmed Death Receptor Ligand 1 and Uses Thereof. State Intellectual Property Office of the Peoples Republic of China (2021). Available at: https://patents.google.com/patent/CN108395443B/en?oq=CN108395443B. CN108395443B.

[70] Wang Y Liwen Z Xin L Wei Y Xiao L Qi Y. Heterocyclic Compound Serving as Pd-L1 Inhibitor. In: World Intellectual Property Organization WO2018196768A1, filed April 25, 2018, and issued November 1, 2018 (2018). Available at: https://patents.google.com/patent/WO2018196768A1/en?oq=WO2018196768.

[71] Yoon KW Byun S Kwon E Hwang S-Y Chu K Hiraki M. Control of Signaling-Mediated Clearance of Apoptotic Cells by the Tumor Suppressor P53. Sci (New York N.Y.) (2015) 349(6247):1261669. doi: 10.1126/science.1261669 PMC5215039 26228159

[72] Sasikumar PGN Ramachandra M Naremaddepalli S. Dual Inhibitors of Vista and Pd-1 Pathways. (2018). Available at: https://patents.google.com/patent/WO2018073754A1/en.

[73] Sasikumar P Sudarshan NS Gowda N Samiulla DS Ramachandra R Chandrasekhar T. Abstract 4861: Oral Immune Checkpoint Antagonists Targeting PD-L1/VISTA or PD-L1/Tim3 for Cancer Therapy. Immunology (2016) 76(14_Supplement)4861. doi: 10.1158/1538-7445.AM2016-4861. American Association for Cancer Research.

[74] Sasikumar PGN Ramachandra M Naremaddepalli SSS Gowda N. Dual Inhibitors of Tim-3 and Pd-1 Pathways. World Intellectual Property Organization. International Bureau WO2019087087A1 (2019). Available at: https://patents.google.com/patent/WO2019087087A1/en?oq=WO2019087087A1.

[75] Sasikumar PG Naremaddepalli SS Ramachandra RK Gowda N Yerramsetti MR Bandireddy SR. “Abstract B006: Functional Antagonism of VSIG8-Mediated Immune Suppression by Oral VISTA Agents.” In: Immune Modulators. American Association for Cancer Research. Philadelphia, PA. (2018). p. B006–6. doi: 10.1158/1535-7163.TARG-17-B006

[76] Sasikumar PG Naremaddepalli SS Ramachandra RK Gowda N Devarapalli S Adurthi S. Abstract 4148: An Orally Bioavailable Small Molecule Antagonist of TIM-3 Signaling Pathway Shows Potent Anti-Tumor Activity. Clin Res (Excluding Clin Trials) (2019) 79(13 Suppl):Abstract nr 4148. doi: 10.1158/1538-7445.AM2019-4148. American Association for Cancer Research.

[77] Sasikumar PGN Ramachandra M Naremaddepalli SS Gundala C. Method of Modulating TIGIT and PD-1 Signalling Pathways Using 1,2,4-Oxadiazole Compounds. (2019). Available at: https://patents.google.com/patent/WO2019175799A2/en?oq=WO2019175799

[78] Sasikumar PGN Ramachandra M Naremaddepalli SSS Gundala C. Compounds as Modulators of TIGIT Signalling Pathway. (2018). Available at: https://patents.google.com/patent/WO2018047139A1/en?oq=WO2018047139

[79] Sasikumar PG Gundala C Balasubramanian WR Naremaddepalli SS Bhumireddy A Patil SS. “Abstract B007: Potent Antitumor Activity of a Novel and Orally Available Small-Molecule Antagonist Targeting the CD47/Sirpα Pathway.” In: Immune Modulators. American Association for Cancer Research. Philadelphia, PA. (2018). p. B007–7. doi: 10.1158/1535-7163.TARG-17-B007

[80] Chupak LS Zheng X. Compounds Useful as Immunomodulators. World Intellectual Property Organization (2015). Available at: https://patents.google.com/patent/WO2015034820A1/en. WO2015034820A1.

[81] Wu Q Jiang L Li S-c He Q-j Yang B Cao J. Small Molecule Inhibitors Targeting the PD-1/PD-L1 Signaling Pathway. Acta Pharmacol Sin (2021) 42(1):1–95. doi: 10.1038/s41401-020-0366-x 32152439 PMC7921448

[82] Blevins DJ Hanley R Bolduc T Powell DA Gignac M Walker K. *In Vitro* Assessment of Putative PD-1/PD-L1 Inhibitors: Suggestions of an Alternative Mode of Action. ACS Med Chem Lett (2019) 10(8):1187–925. doi: 10.1021/acsmedchemlett.9b00221 PMC6691557 31413804

[83] Ganesan A Ahmed M Okoye I Arutyunova E Babu D Turnbull WL. Comprehensive *in Vitro* Characterization of PD-L1 Small Molecule Inhibitors. Sci Rep (2019) 9(1):12392. doi: 10.1038/s41598-019-48826-6 31455818 PMC6712002

[84] Liu M Zhang Y Guo Y Gao J Huang W Dong X. A Comparative Study of the Recent Most Potent Small-Molecule PD-L1 Inhibitors: What Can We Learn? Med Chem Res (2021) 30(6):1230–395. doi: 10.1007/s00044-021-02728-3

[85] Zak KM Grudnik P Guzik K Zieba BJ Musielak B Dömling A. Structural Basis for Small Molecule Targeting of the Programmed Death Ligand 1 (PD-L1). Oncotarget (2016) 7(21):30323–355. doi: 10.18632/oncotarget.8730 PMC5058683 27083005

[86] Krajewski M Rothweiler U D’Silva L Majumdar S Klein C Holak TA. An NMR-Based Antagonist Induced Dissociation Assay for Targeting the Ligand–Protein and Protein–Protein Interactions in Competition Binding Experiments. J Med Chem (2007) 50(18):4382–875. doi: 10.1021/jm070365v 17696513

[87] Musielak B Janczyk W Rodriguez I Plewka J Sala D Magiera-Mularz K. Competition NMR for Detection of Hit/Lead Inhibitors of Protein–Protein Interactions. Molecules (2020) 25(13):30175. doi: 10.3390/molecules25133017 PMC7412237 32630327

[88] Konieczny M Musielak B Kocik J Skalniak L Sala D Czub M. Di-Bromo-Based Small-Molecule Inhibitors of the PD-1/PD-L1 Immune Checkpoint. J Med Chem (2020) 63(19):11271–85. doi: 10.1021/acs.jmedchem.0c01260 PMC7584369 32936638

[89] Sun L Li C-W Chung EM Yang R Kim Y-S Park AH. Targeting Glycosylated PD-1 Induces Potent Anti-Tumor Immunity. Cancer Res (2020) 80(11):2298–10. doi: 10.1158/0008-5472.CAN-19-3133 PMC7272274 32156778

[90] Li C-W Lim S-O Xia W Lee H-H Chan L-C Kuo C-W. Glycosylation and Stabilization of Programmed Death Ligand-1 Suppresses T-Cell Activity. Nat Commun (2016) 7(1):12632. doi: 10.1038/ncomms12632 27572267 PMC5013604

[91] Krajewski M Ozdowy P D’Silva L Rothweiler U Holak TA. NMR Indicates That the Small Molecule RITA Does Not Block P53-MDM2 Binding *in Vitro* . Nat Med (2005) 11(11):1135–365. doi: 10.1038/nm1105-1135 16270059

[92] Grinkevich V Issaeva N Hossain S Pramanik A Selivanova G. Reply to ‘NMR Indicates That the Small Molecule RITA Does Not Block P53-MDM2 Binding *in Vitro* . Nat Med (2005) 11(11):1136–375. doi: 10.1038/nm1105-1136 16270059

[93] Musielak B Kocik J Skalniak L Magiera-Mularz K Sala D Czub M. CA-170 – A Potent Small-Molecule PD-L1 Inhibitor or Not? Molecules (2019) 24(15):28045. doi: 10.3390/molecules24152804 PMC6695792 31374878

[94] Freedberg D Selenko P. Live Cell NMR. Annu Rev Biophys (2014) 43(1):171–925. doi: 10.1146/annurev-biophys-051013-023136 24895852

[95] Siegal G Selenko P. Cells, Drugs and NMR. J Magn Reson (2019) 306(September):202–12. doi: 10.1016/j.jmr.2019.07.018 31358370

[96] Pellecchia M Sem DS Wüthrich K. Nmr in Drug Discovery. Nat Rev Drug Discovery (2002) 1(3):211–195. doi: 10.1038/nrd748 12120505

[97] Shortridge MD Hage DS Harbison GS Powers R. Estimating Protein–Ligand Binding Affinity Using High-Throughput Screening by NMR. J Comb Chem (2008) 10(6):948–585. doi: 10.1021/cc800122m PMC2631241 18831571

[98] Skalniak L Zak KM Guzik K Magiera K Musielak B Pachota M. Small-Molecule Inhibitors of PD-1/PD-L1 Immune Checkpoint Alleviate the PD-L1-Induced Exhaustion of T-Cells. Oncotarget (2017) 8(42):72167–81. doi: 10.18632/oncotarget.20050 PMC5641120 29069777

[99] Liu L Yao Z Wang S Xie T Wu G Zhang H. Syntheses, Biological Evaluations, and Mechanistic Studies of Benzo[c][1,2,5]Oxadiazole Derivatives as Potent PD-L1 Inhibitors With *In Vivo* Antitumor Activity. J Med Chem (2021) 64(12):8391–84095. doi: 10.1021/acs.jmedchem.1c00392 34115499

[100] Powderly J Patel MR Lee JJ Brody J Meric-Bernstam F Hamilton E. CA-170, a First in Class Oral Small Molecule Dual Inhibitor of Immune Checkpoints PD-L1 and VISTA, Demonstrates Tumor Growth Inhibition in Pre-Clinical Models and Promotes T Cell Activation in Phase 1 Study. Ann Oncol (2017) 28:v405–6. doi: 10.1093/annonc/mdx376.007

[101] Radhakrishnan VS Bakhshi S Prabhash K Deshmukh C Nag S Lakshmaiah KC. Phase 2 Trial of CA-170, a Novel Oral Small Molecule Dual Inhibitor of Immune Checkpoints VISTA and PD-1, in Patients (Pts) With Advanced Solid Tumor and Hodgkin Lymphoma. J Immunother Cancer (2018) 6:P714.

[102] Kabelitz D. Antigen-Induced Death of T-Lymphocytes. Front Biosci (1997) 2(4):d61–77. doi: 10.2741/A175 9159213

[103] Rozali EN Hato SV Robinson BW Lake RA Lesterhuis WJ. Programmed Death Ligand 2 in Cancer-Induced Immune Suppression. Clin Dev Immunol (2012) 2012:1–8. doi: 10.1155/2012/656340 PMC3350956 22611421

[104] Yearley JH Gibson C Yu N Moon C Murphy E Juco J. “PD-L2 Expression in Human Tumors: Relevance to Anti-PD-1 Therapy in Cancer.” Clin Cancer Res (2017) 23(12):3158–67. doi: 10.1158/1078-0432.CCR-16-1761 28619999

[105] Shi D An X Bai Q Bing Z Zhou S Liu H. Computational Insight Into the Small Molecule Intervening PD-L1 Dimerization and the Potential Structure-Activity Relationship. Front Chem (2019) 7:764. doi: 10.3389/fchem.2019.00764 31781546 PMC6861162

[106] Bailly C Vergoten G. Protein Homodimer Sequestration With Small Molecules: Focus on PD-L1. Biochem Pharmacol (2020) 174:113821. doi: 10.1016/j.bcp.2020.113821 31972166

[107] Antoni C Vera L Devel L Catalani MP Czarny B Cassar-Lajeunesse E. Crystallization of Bi-Functional Ligand Protein Complexes. J Struct Biol (2013) 182(3):246–545. doi: 10.1016/j.jsb.2013.03.015 23567804

[108] Chen F-F Li Z Ma D Yu Q. Small-Molecule PD-L1 Inhibitor BMS1166 Abrogates the Function of PD-L1 by Blocking Its ER Export. OncoImmunology (2020) 9(1):18311535. doi: 10.1080/2162402X.2020.1831153 PMC7567511 33110706

[109] Li C-W Lim S-O Chung EM Kim Y-S Park AH Yao J. Eradication of Triple-Negative Breast Cancer Cells by Targeting Glycosylated PD-L1. Cancer Cell (2018) 33(2):187–201.e10. doi: 10.1016/j.ccell.2018.01.009 29438695 PMC5824730

[110] Wang L-C Koblish H Zhang Y Kulkarni A Covington M. Incyte - Investors - Events & Presentations (2019). Available at: https://investor.incyte.com/investors/events-and-presentations/default.aspxmodule-scientific-presentations.

[111] Butte MJ Keir ME Phamduy TB Sharpe AH Freeman GJ. Programmed Death-1 Ligand 1 Interacts Specifically With the B7-1 Costimulatory Molecule to Inhibit T Cell Responses. Immunity (2007) 27(1):111–225. doi: 10.1016/j.immuni.2007.05.016 17629517 PMC2707944

[112] Zhao Y Lee CK Lin C-H Gassen RB Xu X Huang Z. PD-L1:CD80 Cis-Heterodimer Triggers the Co-Stimulatory Receptor CD28 While Repressing the Inhibitory PD-1 and CTLA-4 Pathways. Immunity (2019) 51(6):1059–1073.e9. doi: 10.1016/j.immuni.2019.11.003 31757674 PMC6935268

[113] Sugiura D Maruhashi T Okazaki I-m Shimizu K Maeda TK Takemoto T. Restriction of PD-1 Function by *Cis* -PD-L1/CD80 Interactions Is Required for Optimal T Cell Responses. Science (2019) 364(6440):558–66. doi: 10.1126/science.aav7062 31000591

[114] Fenwick C Pellaton C Farina A Radja N Pantaleo G. Identification of Novel Antagonistic Anti-PD-1 Antibodies That Are Non-Blocking of the PD-1/PD-L1 Interaction. J Clin Oncol (2016) 34(15_suppl):3072–2. doi: 10.1200/JCO.2016.34.15_suppl.3072

[115] Scheuplein F Ranganath S McQuade T Wang L Spaulding V Vadde S. Abstract B30: Discovery and Functional Characterization of Novel Anti-PD-1 Antibodies Using *Ex Vivo* Cell-Based Assays, Single-Cell Immunoprofiling, and *in Vivo* Studies in Humanized Mice. Cancer Res (2016) 76(15 Supplement):B30–0. doi: 10.1158/1538-7445.TME16-B30

[116] Piha-Paul S Mitchell T Sahebjam S Mehnert J Karasic T O’Hayer K. 419 Pharmacodynamic Biomarkers Demonstrate T-Cell Activation in Patients Treated With the Oral PD-L1 Inhibitor INCB086550 in a Phase 1 Clinical Trial. J ImmunoTher Cancer (2020) 8(Suppl 3):A445–5. doi: 10.1136/jitc-2020-SITC2020.0419

[117] Kopp WC Smith JW Ewel CH Alvord WG Main C Guyre PM. Immunomodulatory Effects of Interferon-Gamma in Patients With Metastatic Malignant Melanoma. J Immunother (1993) 13(3):181–905. doi: 10.1097/00002371-199304000-00005 8471592

[118] Maluish AE Urba WJ Longo DL Overton WR Coggin D Crisp ER. The Determination of an Immunologically Active Dose of Interferon-Gamma in Patients With Melanoma. J Clin Oncol (1988) 6(3):434–45. doi: 10.1200/JCO.1988.6.3.434 3127550

[119] Talmadge JE Tribble HR Pennington RW Phillips H Wiltrout RH. Immunomodulatory and Immunotherapeutic Properties of Recombinant γ-Interferon and Recombinant Tumor Necrosis Factor in Mice. Cancer Res (1987) 47(10):25635. 3105865

[120] Thompson JA Cox WW Lindgren CG Collins C Neraas KA Bonnem EM. Subcutaneous Recombinant Gamma Interferon in Cancer Patients: Toxicity, Pharmacokinetics, and Immunomodulatory Effects. Cancer Immunol Immunother (1987) 25(1):47–53. doi: 10.1007/BF00199300 3109737 PMC11038648

[121] Sivick KE Desbien AL Glickman LH Reiner GL Corrales L Surh NH. Magnitude of Therapeutic STING Activation Determines CD8+ T Cell-Mediated Anti-Tumor Immunity. Cell Rep (2018) 25(11):3074–3085.e5. doi: 10.1016/j.celrep.2018.11.047 30540940

[122] Lowder JN Freidberg JW Kelly J Freedman AS Coffman R Kanzler H. Dose Finding in Human Trials of TLR9 Agonists: Induction of Interferon-α Inducible Genes in Blood Mononuclear Cells as a Measure of Biologic Activity of 1018 ISS. Blood (2007) 110(11):3844–38445. doi: 10.1182/blood.V110.11.3844.3844

[123] Agrawal S Feng Y Roy A Kollia G Lestini B. Nivolumab Dose Selection: Challenges, Opportunities, and Lessons Learned for Cancer Immunotherapy. J ImmunoTher Cancer (2016) 4(1):725. doi: 10.1186/s40425-016-0177-2 PMC5109842 27879974

[124] Ratain MJ Goldstein DA. Time Is Money: Optimizing the Scheduling of Nivolumab. J Clin Oncol (2018) 36(31):3074–765. doi: 10.1200/JCO.18.00045 30148658

[125] Wang Y Gu T Tian X Li W Zhao R Yang W. A Small Molecule Antagonist of PD-1/PD-L1 Interactions Acts as an Immune Checkpoint Inhibitor for NSCLC and Melanoma Immunotherapy. Front Immunol (2021) 12:654463(May). doi: 10.3389/fimmu.2021.654463 34054817 PMC8160380

